# Effects of a Digital Mental Health Intervention on Perceived Stress and Rumination in Adolescents Aged 13 to 17 Years: Randomized Controlled Trial

**DOI:** 10.2196/54282

**Published:** 2024-03-29

**Authors:** Eliane M Boucher, Haley Ward, Cynthia J Miles, Robert D Henry, Sarah Elizabeth Stoeckl

**Affiliations:** 1 Twill New York, NY United States

**Keywords:** digital intervention, adolescents, adolescent, stress management, mental health, mobile phone, mobile health, mHealth, teen, teens, teenager, teenagers, stress, mental health, rumination, brooding, randomized controlled trial, RCT, randomized, controlled trial, controlled trials, digital mental health intervention, DMHI, digital health

## Abstract

**Background:**

Although adolescents report high levels of stress, they report engaging in few stress management techniques. Consequently, developing effective and targeted programs to help address this transdiagnostic risk factor in adolescence is particularly important. Most stress management programs for adolescents are delivered within schools, and the evidence for these programs is mixed, suggesting a need for alternative options for stress management among adolescents.

**Objective:**

The aim of the study is to test the short-term effects of a self-guided digital mental health intervention (DMHI) designed for adolescents on perceived stress and rumination (ie, brooding).

**Methods:**

This was a 12-week, 2-arm decentralized randomized controlled trial of adolescents aged 13 to 17 years who presented with elevated levels of perceived stress and brooding. Participants were randomly assigned to engage with a self-guided DMHI (Happify for Teens) or to a waitlist control. Participants assigned to the intervention group were given access to the program for 12 weeks. Happify for Teens consists of various evidence-based activities drawn from therapeutic modalities such as cognitive behavioral therapy, positive psychology, and mindfulness, which are then organized into several programs targeting specific areas of concern (eg, Stress Buster 101). Participants in the waitlist control received access to this product for 12 weeks upon completing the study. Participants in both groups completed measures of perceived stress, brooding, optimism, sleep disturbance, and loneliness at baseline, 4 weeks, 8 weeks, and 12 weeks. Changes in outcomes between the intervention and waitlist control groups were assessed using repeated-measures multilevel models.

**Results:**

Of the 303 participants included in data analyses, 132 were assigned to the intervention and 171 to the waitlist. There were significantly greater improvements in the intervention condition for perceived stress (intervention: *B*=–1.50; 95% CI –1.82 to –1.19; *P*<.001 and control: *B*=–0.09; 95% CI –0.44 to 0.26; *P*=.61), brooding (intervention: *B*=–0.84; 95% CI –1.00 to –0.68; *P*<.001 and control: *B*=–0.30; 95% CI –0.47 to –0.12; *P*=.001), and loneliness (intervention: *B*=–0.96; 95% CI –1.2 to –0.73; *P*<.001 and control: *B*=–0.38; 95% CI: –0.64 to –0.12; *P*=.005) over the 12-week study period. Changes in optimism and sleep disturbance were not significantly different across groups (*P*s≥.096).

**Conclusions:**

Happify for Teens was effective at reducing perceived stress, rumination, and loneliness among adolescents over 12 weeks when compared to a waitlist control group. Our data reveal the potential benefits of DMHIs for adolescents, which may present a more scalable, destigmatized, and cost-effective alternative to school-based programs.

**Trial Registration:**

ClinicalTrials.gov NCT04567888; https://clinicaltrials.gov/ct2/show/NCT04567888

**International Registered Report Identifier (IRRID):**

RR2-10.2196/25545

## Introduction

### Background

The onset of many mental health disorders occurs in childhood and adolescence, and these disorders often continue into adulthood [1,2]. Already, approximately 20% of adolescents worldwide have at least 1 mental health disorder [3]; however, rates of mental illness among adolescents are increasing [4] and at steeper rates than among adults [5]. Addressing mental health in adolescence is critical to reducing the prevalence and consequences of mental health disorders in adulthood.

Despite public health efforts to increase access to mental health services for youths, the use of these services by adolescents with mental health disorders remains low [6]. Research suggests that only 50% of US adolescents with mental illness seek treatment [7], and as few as 25% actually receive treatment [8]. Paradoxically, national trends indicate that, overall, the use of outpatient mental health services by US adolescents, including both psychotherapy and psychotropic medications, has increased over time [9]. However, this increase is primarily attributed to adolescents with less severe or no mental health impairment [9]. This is problematic, given the shortage of mental health service providers for children and adolescents, particularly in rural and low-income areas [10]. The increased use of these services by adolescents likely places a strain on the available services for youths and, in turn, adolescents, with more serious mental health disorders may have difficulties receiving necessary treatment. Interventions that are focused on promoting flourishing and preventing future dysfunction and impairment may help to reduce the burden on mental health services by offering alternative services to adolescents with little or no mental health impairment [11].

### Stress as a Risk Factor in Adolescence

Adolescence is a developmental stage where individuals may be particularly sensitive to stress due to shifts in hypothalamic-pituitary-adrenal axis reactivity, leading to more intense hormonal responses to stressors, particularly in older adolescents [12]. Over the last decade, researchers have become increasingly interested in adolescent stress as an important transdiagnostic risk factor. Although animal models suggest that predictable, chronic mild stress levels in adolescence can actually increase resilience in adulthood [13], research suggests many adolescents are coping with more severe stress levels. In one study, 22% of adolescents aged 15 to 17 years reported moderate to severe levels of perceived stress [14]. In addition, national surveys conducted by the American Psychological Association [15,16] have found that adolescents report higher levels of stress than most adults, particularly during the school year.

Such high levels of stress have been linked to lower life satisfaction [17], poorer academic performance [18,19], cigarette smoking [20], emotional eating [21], poorer diet [22], more frequent subjective health complaints (eg, headaches, fatigue, and sleep difficulties) [23], as well as internalizing symptoms including depression [24,25] and anxiety [25]. Interpersonal stress, in particular, predicts the onset of a first major depressive episode among adolescents [26]. Thus, addressing stress in adolescents is critical to avoiding more long-term problems.

However, 25% of adolescents self-report not doing enough to manage their stress [16]. Approximately 55% of adolescents report setting aside time for stress management “a few times a month or less,” 13% report never setting aside time for stress management, and just 5% report having seen a mental health professional about their stress [15]. Based on these data, the American Psychological Association noted the need for opportunities to help adolescents address and cope with their stress in order “to break this unhealthy legacy of stress in America” [15].

Although there has been an increase in interventions targeting adolescent stress [27], evidence-based interventions for stress are much less common than those for anxiety and depression [28]. Furthermore, most interventions for stress are school-based, and the evidence for these interventions is mixed. In one systematic review of stress management interventions, only 58% of the reviewed studies found significant improvements in physiological indicators of stress (eg, blood pressure) or self-reported stress [29]. More recent meta-analyses of school-based programs further suggest that these programs may only be effective for school rather than social stress [27]. Given these findings, we need more research exploring the effectiveness of stress management programs with adolescents, particularly those that could be delivered outside schools.

### Negative Cognitions and Stress

In addition, given that stress in adolescence stems, at least in part, from feelings of helplessness and negative affect [30], effective interventions for adolescent stress may need to target underlying negative cognitions as well as perceived stress. However, recommended approaches to addressing stress tend to focus on stress management training, relaxation training, and problem-solving skill or decision-making skill training [27,28,31], which may not adequately address the related negative cognitions and cognitive processes.

Indeed, there is evidence that improving the content of adolescents’ cognitions can help attenuate the consequences of stress. For example, interpersonal stress predicts depressive symptoms in adolescents only when coupled with negative cognitions [26] or persistent low positive affect [32], whereas self-compassion buffers against the negative consequences of stress on internalizing symptoms [25]. In other words, addressing both negative cognitions and stress together may be particularly important to mitigate the negative consequences of stress [26].

Rumination—a pattern of repeatedly thinking about the causes, consequences, and symptoms of one’s negative affect [33]—is one cognitive process that may be particularly relevant to stress and future risk of mental health concerns. Because rumination can occur in response to stressful events, it also plays a role in the negative consequences of chronic stress. Longitudinal research has shown that more frequent stressful life events predict increased rumination among both adults and adolescents, and that rumination mediates the relationship between stressful life events and anxiety symptoms in adolescents [34]. Other research has shown that adolescents who ruminate in response to stress are at greater risk of depression and substance misuse [35].

Rumination may also increase perceived stress. In one study, rumination exacerbated the relationship between life hassles and depression, anxiety, and stress in adolescents [36]. Other research shows that rumination increases adolescents’ likelihood of experiencing interpersonal stress which, in turn, predicts more internalizing symptoms [37]. Therefore, applying approaches that are more traditionally applied to addressing negative cognitive processes like rumination, such as cognitive restructuring, self-monitoring, acceptance strategies, and attention control training [31], may be helpful in reducing stress as well.

These findings suggest there may be a common pathway to reducing both rumination and perceived stress in adolescents. Therefore, identifying interventions that can address both would be beneficial as they would target 2, rather than 1, important transdiagnostic risk factors in the adolescent population.

### Objectives

The aim of this study was to test the impact of a digital mental health intervention (DMHI) designed for adolescents called Happify for Teens (Twill Inc). This program has been adapted for adolescents between 13 and 17 years of age from another DMHI developed for adults (Happify; Twill Inc). Happify for Teens consists of gamified versions of evidence-based activities adapted from various therapeutic approaches including cognitive behavioral therapy (CBT) [38], mindfulness-based stress reduction [39], and positive psychology [40,41]. Consequently, these programs integrate a variety of recommended approaches to addressing negative cognitions [31] that also help manage stress [42-45].

Observational studies of real-world Happify users have shown significant improvements in subjective well-being and anxiety among adults after 6-8 weeks of use, with greater gains among users who completed more activities [46-48]. Randomized controlled trials (RCTs) with adult participants have similarly shown improvements in depressive symptoms, anxiety, and resilience among those who completed at least 2 activities per week relative to a control group [49,50]. However, Happify for Teens has yet to be tested empirically. Therefore, in this study, we recruited adolescents aged 13 to 17 years who reported elevated stress and rumination. Participants were randomly assigned to have access to Happify for Teens for 12 weeks or to a corresponding waitlist control group. Changes in perceived stress after 4, 8, and 12 weeks across the 2 groups were compared. We also examined changes in brooding (the maladaptive component of rumination) as a secondary outcome and in sleep disturbance, loneliness, and optimism as exploratory outcomes.

## Methods

### Study Design

This was a 12-week, 2-arm RCT of adolescents aged 13 to 17 years residing in the United States with elevated levels of perceived stress and brooding (Multimedia Appendix 1).

### Ethical Considerations

A study protocol was previously published [51], and the trial was registered with ClinicalTrials.gov (NCT04567888). Study procedures were reviewed and approved by IntegReview (HLS-20), an independent institutional review board, which was acquired by Advarra after the study was launched. Parental consent was obtained electronically as part of the prescreening questionnaire, and participant assent was obtained electronically before participants began the baseline assessment, after qualifying for the study. The use of e-signatures was approved by IntegReview. Participants received US $20 for completing each of the 4-, 8-, and 12-week assessments and a US $20 bonus if they completed all 4 assessments. Participants were not compensated for engaging in the intervention. All data were deidentified for analysis, and responses to the baseline, 4-, 8-, and 12-week assessments were matched using a user-generated ID code, which was entered at the beginning of each assessment.

### Participants

The study was advertised to parents or guardians via social media, emails to Happify users, within US schools, and using a snowballing recruitment method. Parents or guardians were directed to complete a prescreening questionnaire to report their child’s age, country of residence, and previous Happify use and to provide electronic consent for each eligible child. Adolescents who were qualified based on the prescreening questionnaire were then invited to complete a screening questionnaire via email; adolescents were eligible to participate if they reported being 13 to 17 years of age, residing in the United States, having never used Happify, and reporting elevated stress (Perceived Stress Scale [52] scores >14) and brooding (Ruminative Response Scale—Short Form—Brooding Subscale scores [53] ≥10).

### Procedure

Eligible participants were directed to complete the baseline assessment and provide electronic assent to participate in the study. To enroll in the study, participants had to provide electronic assent, complete the baseline assessment, and pass at least 1 attention check.

Once enrolled, participants were randomized to condition; randomization was completed automatically by Qualtrics (Qualtrics) using block randomization upon completing the baseline assessment and meeting final eligibility criteria. Those assigned to the intervention condition received instructions to download the Happify mobile app and were directed to the Happify for Teens platform after creating an account. Like the original Happify program, Happify for Teens consists of digital versions of evidence-based activities, which are categorized into 6 different skills: *Savor* (activities focused on developing mindfulness), *thank* (activities focused on gratitude), *Aspire* (activities focused on optimism, goal-setting, and finding meaning and purpose), *Give* (activities focused on acts of kindness, forgiveness, or promoting prosocial behavior), *Empathize* (activities fostering self-compassion and perspective-taking), and *Revive* (activities focused on physical health). In Happify for Teens, these activities were modified and then reviewed by a panel of adolescents to ensure the language and content were both appropriate and relevant. As in the original Happify program, activities are organized into “tracks,” 4-week programs intended to address a specific area of concern, like increasing one’s confidence or reducing stress. Users are able to switch tracks at any time and can also access activities outside these tracks, on demand, through the Instant Play feature. More information on the Happify for Teens program, including screenshots, can be found elsewhere [51].

Participants were given access to all Happify for Teens tracks; however, the same track (*Stop the Worry Cycle*) was shown as the featured track for all participants and recommended upon signing up for the program. Participants received no instructions on how much to engage with the intervention but received push notifications and weekly engagement emails as part of the intervention. We also contacted participants who completed no activities over 7 days via email or SMS text message (based on participant preference). Participants had access to the intervention for the full study period but stopped receiving push notifications or emails regarding use after 8 weeks; this was done to provide a period of time where engagement with the program may be more naturalistic, given that RCT procedures tend to be associated with inflated engagement with DMHIs [54]. Participants in both conditions also received regular emails or SMS text messages (depending on participant preference) about their progress to boost retention. These emails were identical regardless of condition and reminded participants of an upcoming assessment (when appropriate) or asked if the participant had any concerns. In total, 5 such emails were sent throughout the 12-week study period. Waitlist participants received access to the intervention after 12 weeks.

Every 4 weeks, participants were prompted to complete outcome measures via Qualtrics. Participants were contacted via email or SMS text message (based on participant preference) if an assessment was not completed within 7 days of its scheduled date.

### Measurements and Outcomes

#### Primary Outcome: Perceived Stress

The Perceived Stress Scale [52] consists of 10 items asking participants how often they felt each feeling or thought in the previous month (eg, “How often have you felt nervous and ‘stressed’?”). Items are rated on a scale from 0 (never) to 4 (very often), and ratings are summed, so higher scores indicate greater perceived stress. Across the 4 time points, internal reliabilities ranged from acceptable to good (αs=.77 to .88).

#### Secondary Outcome: Brooding

The Brooding Subscale of the Ruminative Response Scale [53] asks respondents to indicate how often they engage in 5 behaviors (eg, “Think ‘Why can’t I handle things better’?”) on a scale from 1 (almost never) to 4 (almost always). Participants in this study were asked to reflect on the previous month. Ratings are summed, so higher scores indicate more brooding. Internal reliability values were fair to acceptable (αs=.61 to .78).

#### Exploratory Outcomes

##### Sleep Disturbance

The PROMIS (Patient-Reported Outcomes Measurement Information System) Pediatric Sleep Disturbance Scale—Short Form 4a [55] measures the extent to which participants experienced sleep disturbances over the previous 7 days (eg, “In the past 7 days, I had trouble sleeping”). Items are rated on a scale from 1 (never) to 5 (always), and ratings are summed, so higher scores indicate more sleep disturbance. Internal consistency ranged from good to excellent (αs=.89 to .91).

##### Loneliness

The Roberts UCLA Loneliness Scale [56] consists of 8 items (eg, “I lack companionship”). Respondents rate each statement on a 4-point scale from 0 (never) to 3 (often), and ratings are summed, so higher scores indicate greater loneliness. Internal reliability values ranged were all good (αs=.82 to .85).

##### Optimism

The Life Orientation Test—Revised [57] consists of 10 items (eg, “I’m always optimistic about my future”). Respondents indicate their level of agreement with each statement on a 5-point scale from 0 (strongly disagree) to 4 (strongly agree). After dropping 4 filler items, ratings are summed, so higher scores indicate more optimism. Internal reliabilities were acceptable (αs=.72 to .77).

### Statistical Analysis

To assess whether changes in outcomes differed across groups, we created repeated-measures multilevel models (MLMs) for each outcome. MLMs were computed in R software (R Foundation for Statistical Computing) using the *lme4* package [58] and restricted maximum likelihood estimation. All models included a random slope for time (Δ*χ*^2^_2_s≥10.3; *P*s≤.01) and cross-level interactions for condition (waitlist and intervention)×time (baseline, 4 weeks, 8 weeks, and 12 weeks). We examined regression assumptions (eg, residual normality and influential observations), and final models were run with the small number of influential outliers removed (2-4 observations, depending on the outcome). Note that these analyses differ from those in the published protocol [51] because data violated the sphericity assumption for repeated-measures ANOVAs. Additionally, MLMs can manage missing outcome data without imputation, allowing us to retain more participants. Due to power constraints, we focus only on analyses testing changes in primary, secondary, and exploratory outcomes across conditions.

In addition, because careless responding is problematic with web-based surveys and may artificially increase relationships between variables, we used two a priori mechanisms for identifying low-quality data: (1) failing 3 or more attention checks in any given assessment and (2) completing an assessment at a rate faster than 1 second per item [59]. Specific assessments that met either of these criteria were dropped rather than dropping the participant altogether, and analyses were rerun without these assessments as a sensitivity analysis.

## Results

### Sample

Study enrollment began on June 3, 2021, and continued until December 18, 2021. Overall, 2332 parents or guardians completed the screening questionnaire, of which 1604 met initial inclusion criteria and provided consent. In total, 631 adolescents proceeded to complete the screener, of which 353 met inclusion criteria, completed the baseline assessment, and were enrolled into the study and assigned to condition (Happify for Teens: n=178 and waitlist: n=175). However, 22 participants in the intervention condition did not create a Happify for Teens account and were withdrawn from the study.

Note that this sample is smaller than the target sample size reported in the published protocol [51]. As described in that protocol, however, power analyses indicated that 200 participants would be sufficient for 80% power to detect a small effect for our primary outcome. Due to the high levels of dropout observed with digital interventions [60], including with adolescents, we assumed a 75% attrition rate and set a target sample size of 800 participants. Because attrition rates were much lower than expected (assessment completion rates ranged from 78.95% to 87.13%), we decided to stop enrollment once 100 participants per condition completed the 12-week assessment to avoid oversampling, resulting in a smaller sample.

### Data Exclusion

**Figure 1:**
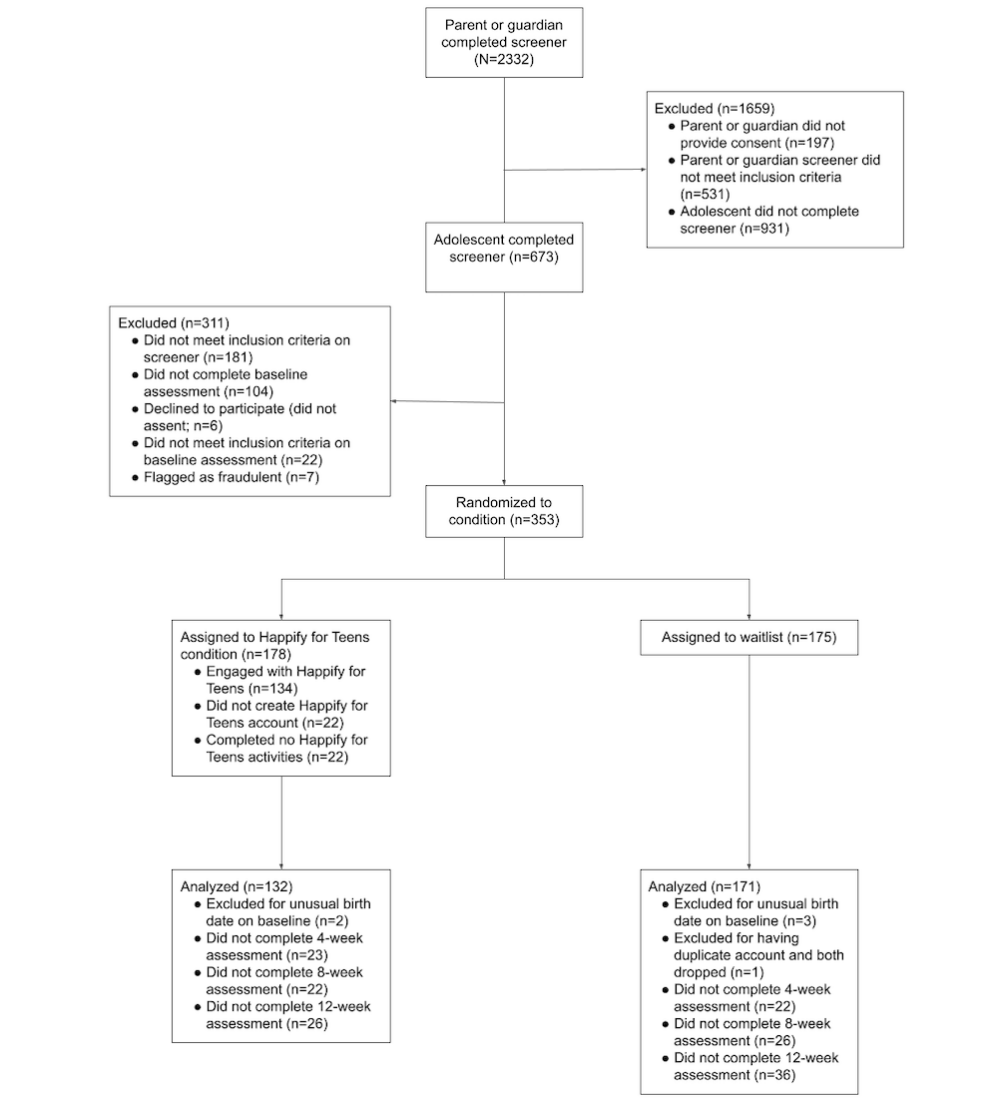
Flow of the participants in the randomized controlled trial.

Of the 331 participants who completed the study, 28 were excluded from data analyses (Figure 1). Of note, 22 participants in the intervention condition failed to complete any activities throughout the study period (and thus were not exposed to the intervention). In total, 5 participants were excluded for providing odd or missing birth years on the baseline assessment, and 1 additional participant was excluded for having duplicate responses to assessments. This resulted in a total of 303 participants included in our analyses (Happify for Teens: n=132 and waitlist n=171). Because the failure to engage with the intervention is subject to self-selection bias, and thus excluding these participants from the analysis means the sample is not fully randomized, we also conducted sensitivity analyses including participants who signed up for the program but completed no activities (n=22) as well as those participants randomized to the Happify for Teens condition who never created an account on the platform (n=22).

### Sample Demographics

Sample demographics are presented in detail in Table 1. The majority of the sample identified as female (n=202, 66.7%) and as White (n=262, 86.5%). Fewer participants reported being 13 years of age relative to other age groups, but all qualifying age groups were represented in our sample. When starting the study, the majority of participants (n=226, 74.6%) were enrolled in school full-time, in person.

**Table 1:** Demographic characteristics for analytical sample.

			Total sample (N=303), n (%)		Happify for Teens (n=132), n (%)		Waitlist (n=171), n (%)			
**Gender^a^**										
	Female	202 (66.7)		94 (71.2)		108 (63.2)				
	Male	76 (25.1)		27 (20.4)		49 (28.6)				
	Neither	24 (7.9)		11 (8.3)		13 (7.6)				
	Not reported	1 (0.003)		0 (0)		1 (0.6)				
**Age^b^ (years)**										
	13	38 (12.5)		14 (10.6)		24 (14)				
	14	75 (24.7)		33 (25)		42 (24.6)				
	15	53 (17.5)		22 (16.7)		31 (18.1)				
	16	69 (22.8)		27 (20.4)		42 (24.6)				
	17	68 (22.4)		36 (27.3)		32 (18.7)				
**Schooling situation**										
	Not attending school	6 (2)		2 (1.5)		4 (2.3)				
	Attending school, in person	226 (74.6)		97 (73.5)		129 (75.4)				
	Attending school, virtual	22 (7.3)		8 (6.1)		14 (8.2)				
	Attending school, hybrid	22 (7.3)		13 (9.8)		9 (5.3)				
	Homeschooled	27 (8.9)		12 (9.1)		15 (8.8)				
**Race^c^**										
	American Indian or Alaska Native	9 (3)		4 (3)		5 (2.9)				
	Asian or Asian American	14 (4.6)		10 (7.6)		4 (2.3)				
	Black or African American	32 (10.6)		10 (7.6)		22 (12.9)				
	Hispanic or Latinx	33 (10.9)		12 (9.1)		21 (12.3)				
	Native Hawaiian, other Pacific Islander	2 (0.7)		0 (0)		2 (1.2)				
	White	262 (86.5)		117 (88.6)		145 (84.8)				
	Other	9 (3)		3 (2.3)		6 (3.5)				
**Current use of wellness apps**										
	Yes	35 (11.6)		15 (11.4)		20 (11.7)				
	No	268 (88.4)		117 (88.6)		151 (88.3)				

^a^Gender was assessed using an open-ended question (What is your gender?); responses clearly identifying male or female were coded as such, and other responses were combined into the “neither” category.

^b^Age was estimated using participants’ reported birth year at baseline. Adolescents reported their age during screening, and those reporting ages 13 years and younger or 17 years and older were disqualified. Nevertheless, some participants may have turned 18 years before the baseline assessment or during the study period.

^c^Race was a multioption question, consequently percentages across categories sum to more than 100%.

Most participants were not currently using any self-care or wellness apps (n=268, 88.4%). Among those currently using these programs, there was a great deal of variability in programs, but *I am Sober* (n=4) and *Calm* (n=3) were reported most frequently. Additionally, although we used current Happify members as one recruitment mechanism, few participants reported their parents used Happify (n=25, 8.3%).

### Happify for Teens Use

Of the participants assigned to the Happify for Teens condition who signed up for the program, 85.7% (132/154) completed at least 1 activity over the 12-week study period. The mean number of activities completed among these participants was 46.24 (SD 53.02), ranging from 1 to 235 activities.

Consistent with other DMHI research, engagement dropped over the course of the 12-week study period. More specifically, among participants who completed at least 1 activity, 3 (2.3%) completed no activities during the first 4 weeks, whereas 25 (18.9%) completed no activities between week 5 and week 8, and 60 (45.5%) completed no activities between week 9 and week 12. Similarly, mean levels of engagement dropped over time, with an average of 22.49 (SD 22.29) activities completed between week 1 and week 4 compared to an average of 15.25 (SD 21.11) activities completed between week 5 and week 8 and 8.5 (SD 14.49) activities completed between week 9 and week 12.

Although participants were not given explicit instructions on how often to engage with the Happify for Teens platform or how many activities to complete, previous research with adults suggests that completing an average of 2 or more activities is optimal [49,50,61,62]. Among participants who completed at least 1 activity, 75.8% (n=100) engaged at the optimal level during the first 4 weeks, 50% (n=66) engaged at the optimal level between week 5 and week 8, and 29.5% (n=39) engaged at the optimal level between week 9 and week 12. These levels are consistent or better than what has been observed in previous research using adults [49,61,62].

### Changes in Outcomes

#### Overview

Descriptive statistics for each outcome by assessment period are presented in Table 2. All final MLMs reported below included a random slope for time (Δ*χ*^2^_2_s≥10.3; *P*s≤.01). Only 4 participants were flagged for attention checks (1 on 3 of 4 assessments, 1 on 2 assessments, and 2 on just 1 assessment). No participants were flagged for completion rate. Primary and secondary outcomes did not change meaningfully in these analyses when these assessments were excluded. In addition, outcomes did not change meaningfully in sensitivity analyses including participants who had been randomly assigned to the intervention condition but never engaged with the Happify for Teens program. More specifically, no main effects of time or condition changed for any of the primary, secondary, or exploratory outcomes nor did any of the time×condition interactions. However, some nonsignificant effects for control variables did become significant in the sensitivity analyses. The effect of age became significant for perceived stress (*P*=.04), such that stress increased with age. The effect of gender also became significant for both optimism (*P*=.03) and loneliness (*P*=.02), such that adolescents who identified as male reported higher levels of optimism but also higher levels of loneliness compared to other gender identities.

**Table 2:** Descriptive statistics for all outcomes by assessment period and condition.

			α		Total sample, mean (SD)		Happify for Teens, mean (SD)		Waitlist control, mean (SD)
**Perceived stress^a^**									
	Baseline	.77		25.73 (4.77)		26.33 (4.43)		25.26 (4.97)	
	4 weeks	.84		23.55 (5.82)		22.01 (6.10)		24.67 (5.36)	
	8 weeks	.86		23.47 (6.27)		21.95 (6.20)		24.61 (6.10)	
	12 weeks	.88		22.91 (6.62)		20.96 (6.57)		24.44 (6.27)	
**Brooding^b^**									
	Baseline	.61		14.29 (2.47)		14.29 (2.36)		14.29 (2.56)	
	4 weeks	.75		13.32 (3.18)		12.53 (3.24)		13.89 (3.01)	
	8 weeks	.72		12.65 (2.93)		11.94 (2.89)		13.20 (2.85)	
	12 weeks	.78		12.68 (3.22)		11.68 (3.18)		13.46 (3.03)	
**Sleep disturbance** ^c^									
	Baseline	.89		12.60 (3.87)		12.51 (4.01)		12.67 (5.04)	
	4 weeks	.91		11.88 (4.07)		11.32 (4.10)		12.29 (4.01)	
	8 weeks	.90		11.46 (3.92)		10.86 (3.63)		11.92 (4.09)	
	12 weeks	.90		11.31 (4.15)		10.88 (3.76)		11.65 (4.42)	
**Loneliness^d^**									
	Baseline	.83		21.15 (4.93)		21.25 (4.80)		21.06 (5.04)	
	4 weeks	.82		20.29 (4.82)		19.69 (5.15)		20.72 (4.52)	
	8 weeks	.85		19.62 (5.08)		19.14 (5.33)		19.98 (4.87)	
	12 weeks	.82		19.09 (4.68)		18.05 (4.98)		19.91 (4.28)	
**Optimism^e^**									
	Baseline	.72		10.31 (3.40)		9.99 (3.40)		10.56 (3.39)	
	4 weeks	.77		10.89 (3.60)		10.80 (3.64)		10.95 (3.57)	
	8 weeks	.77		11.05 (3.70)		11.21 (3.76)		10.93 (3.67)	
	12 weeks	.75		11.41 (3.70)		11.42 (3.76)		11.39 (3.66)	

^a^Perceived stress was measured with the Perceived Stress Scale [52].

^b^Brooding was measured using the Ruminative Response Scale—Brooding Subscale [53].

^c^Sleep disturbance was measured using the PROMIS (Patient-Reported Outcomes Measurement Information System) Pediatric Sleep Disturbance Scale—Short Form 4a [54].

^d^Loneliness was measured using the Roberts UCLA Loneliness Scale [55].

^e^Optimism was measured using the Life Orientation Test—Revised [56].

#### Primary Outcome: Perceived Stress

**Figure 2:**
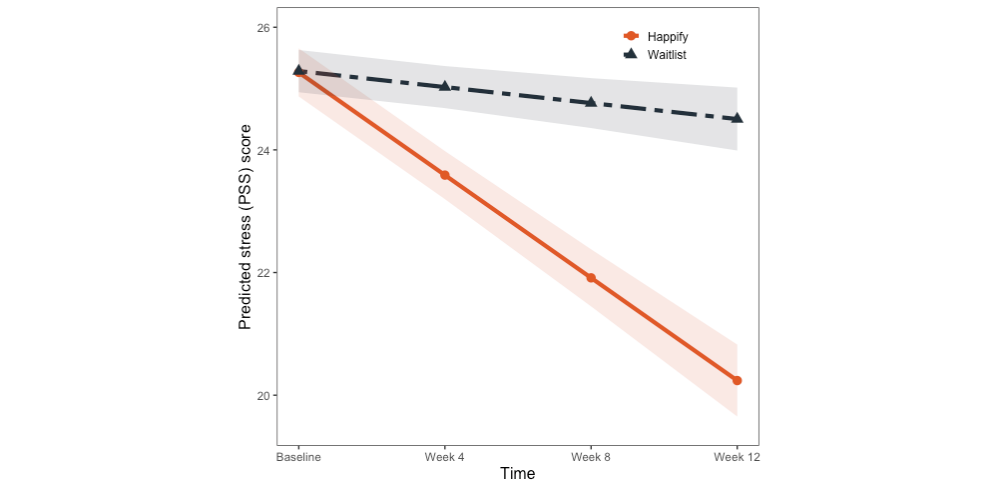
Model-predicted changes in perceived stress scores across time and condition. PSS: Perceived Stress Scale.

We found a significant main effect of time (*P*<.001) but no significant effect of condition (*P*=.97). These were qualified by the predicted condition×time interaction (*P*<.001). To break down this interaction, we calculated simple slopes for each condition, which revealed a significant reduction in perceived stress among participants in the intervention condition (*B*=–1.50; 95% CI –1.82 to –1.19; *P*<.001) but not among those in the waitlist control (*B*=–0.09; 95% CI –0.44 to 0.26; *P*=.61; Figure 2). With the exception of a significant effect of gender (*P*<.001) suggesting that participants identifying as male had significantly lower PSS scores overall, no other effects were significant (*P*s≥.06).

#### Secondary Outcome: Brooding

**Figure 3:**
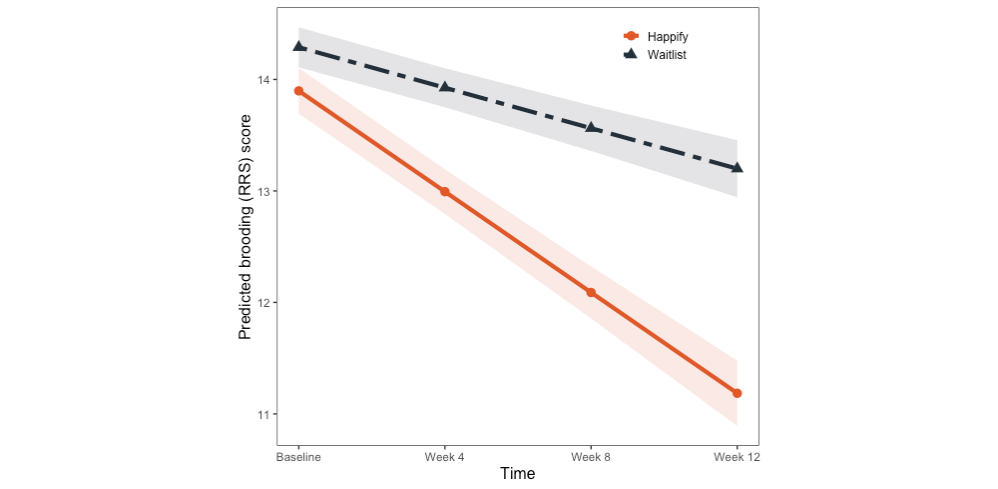
Model-predicted changes in RRS—Brooding Subscale scores across time and condition. RRS: Ruminative Response Scale.

We also found a significant effect of time (*P*<.001) but not of condition (*P*=.15) for brooding. These effects were qualified by a significant condition×time interaction (*P*<.001). There were significant reductions in brooding among participants in the waitlist control (*B*=–0.30; 95% CI –0.47 to –0.12; *P*=.001) as well as those in the Happify for Teens condition (*B*=–0.84; 95% CI –1.00 to –0.68; *P*<.001); however, CIs for the 2 groups did not overlap, suggesting that improvements were relatively greater among those in the intervention condition (Figure 3). Again, with the exception of a significant effect of gender suggesting that participants identifying as male reported significantly lower levels of brooding (*P*<.001), no other effects were significant (*P*s≥.35).

#### Exploratory Outcomes

For sleep disturbance and optimism, we found a significant effect of time (*P*s<.001) and no significant effect of condition (*P*s≥.28). However, the corresponding condition×time interactions were not significant (*P*s≥.10), suggesting that while there were improvements in sleep disturbance and optimism over time, improvements did not differ by condition. We did find a significant effect of age for sleep disturbance (*P*=.04), suggesting that sleep disturbance was greater as age increased, but no other effects were significant (*P*s≥.11). When assessments failing the attention check criterion were dropped, the condition×time interaction did become significant for sleep disturbance (*P*=.04). Because no other outcomes changed substantially in the sensitivity analyses, we did not interpret this interaction to avoid making a type I error.

**Figure 4:**
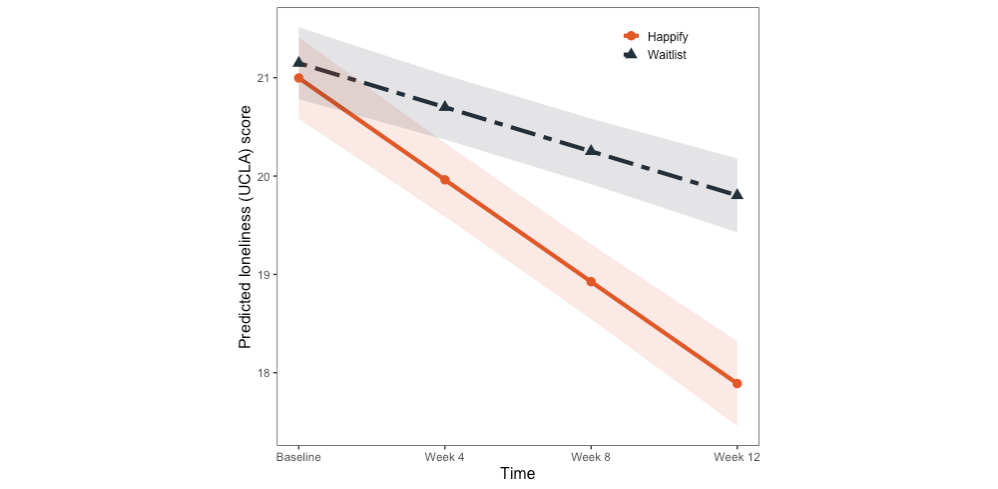
Model-predicted changes in UCLA loneliness scores across time and condition.

For loneliness, we also found a significant effect of time (*P*<.001) and no significant effect of condition (*P*=.79), but the corresponding condition×time interaction was significant (*P*<.001). As with perceived stress and brooding, simple slopes for each condition were calculated, which revealed significant reductions in Roberts UCLA Loneliness Scale-8 scores among participants in the waitlist control (*B*=–0.38; 95% CI –0.64 to –0.12; *P*=.005) as well as those in the intervention condition (*B*=–0.96; 95% CI –1.2 to –0.73; *P*<.001). Similar to changes in brooding, however, the CIs for the 2 groups did not overlap, suggesting that improvements in loneliness were greater in the Happify for Teens group than in the waitlist control (Figure 4). No other effects were significant (*P*s≥.16).

## Discussion

### Principal Findings

The purpose of this study was to test the efficacy of a DMHI designed for adolescents on perceived stress and brooding. Our findings suggest that adolescents who were engaged with the intervention had significant reductions in perceived stress, rumination, and loneliness over 12 weeks relative to those in a waitlist control but not in sleep disturbance or optimism.

Previous DMHI research with adolescents has focused primarily on depression and anxiety. For instance, in a recent review of 18 meta-analyses and systematic reviews of digital health interventions for adolescents [63], only 3 included stress as an outcome. Consistent with the present findings, these studies found significant improvements in stress among adolescents engaging with a digital intervention, which suggests DMHIs can be effective in improving stress management among adolescents. To our knowledge, however, this study was the first to explore the effects of a digital intervention on rumination among adolescents. Given that rumination predicts future dysfunction including anxiety, depression, and substance use [34,35], intervening early to reduce the frequency of brooding is another important mechanism for prevention.

We also found that the intervention significantly improved participants’ loneliness but not optimism or sleep disturbance. Although these were exploratory outcomes, the effect on loneliness is not unexpected, given that Happify for Teens includes information targeting loneliness. Research conducted on the adult version of Happify also suggests that users perceive several activities that appear in the adolescent version as helpful for addressing their feelings of loneliness [64]. In contrast, sleep is not a focus within the program; and, consequently, to impact sleep outcomes, a more targeted intervention may be necessary.

The reasons why we did not find an effect on optimism are less clear. One potential explanation is that the Life Orientation Test—Revised is a measure of dispositional optimism and thus may be less sensitive to changes in optimism over time. Indeed, a recent study suggested that a state measure of optimism may be more suitable for assessing within-person changes [65]. Another possibility is that the study was conducted during the COVID-19 pandemic, which may have impacted participants’ levels of optimism and the ability of the intervention to impact optimism. Consequently, additional research is needed to better understand whether this particular intervention can also improve optimism as well as perceived stress, brooding, and loneliness.

### Leveraging DMHIs for Mental Health Support in Adolescence

Given that rates of mental illness among adolescents are increasing [4], and at a steeper rate than among adults [5], identifying opportunities to increase access to mental health care in this population is critical. Currently, most stress management programs for adolescents continue to be delivered within schools [27-29]. Although school-based prevention programs can be effective [28,29], there are numerous barriers to implementing evidence-based interventions within schools, including lack of time and resources and financial constraints [66]. Costs associated with implementing such interventions may be particularly prohibitive in socially and economically disadvantaged areas [28], where students may need these interventions most [27]. Even when such programs can be implemented, student participation may be negatively impacted by fears of stigmatization [67]. Given that 95% of US adolescents own, or have access to, a cellular phone, and 88% have daily access to a computer [68], digital interventions may offer a better, and arguably more cost-effective, opportunity to reach more adolescents while potentially reducing concerns with stigma by increasing privacy and confidentiality.

DMHIs have become increasingly popular [69], and although interventions developed specifically for youths are still relatively scarce [70], interest in developing digital tools specifically for adolescents is also increasing. For example, Woebot Health has developed an intervention for adolescents with mild to moderate depression and anxiety that uses a relational agent to deliver CBT, interpersonal psychotherapy for adolescents, and elements of dialectical behavior therapy. Although results are not yet published, they recently completed an RCT testing this DMHI (ClinicalTrials.gov NCT05486611). Similarly, Limbix developed SparkRx, a DMHI drawing on behavioral activation and CBT designed for adolescents with depression, which has been shown to significantly improve symptoms of depression among adolescents who were engaged consistently with the intervention [71]. However, both interventions have been developed for adolescents with clinical levels of depression, which offer important support to adolescents struggling with mental illness but do not address the increased burden on mental health services coming from adolescents with less severe or no mental health impairment.

### Limitations and Future Directions

One limitation of this design is the lack of an active control group. Although the inclusion of a waitlist control accounts for some threats to internal validity (eg, regression to the mean and maturation), we cannot rule out placebo effects. In particular, some researchers argue there are unique considerations for placebo effects in the context of digital interventions, including participants’ beliefs about technology and how the mobile app is designed [72]. Research has shown that the impact of digital interventions is typically weaker when compared to active control, though these interventions still appear to outperform attentional controls in terms of depression and anxiety [73]. Previous research conducted on the adult version of Happify also showed superior effects on depression, anxiety, and resilience compared to an active control [49,50]. Nevertheless, additional research comparing the effects of Happify for Teens to a digital sham condition will be important to rule out any potential placebo effects.

Another limitation of this study was the relative homogeneity of participants, particularly in terms of race and schooling situation. Specifically, our sample was predominantly White and attended school full-time in person. Moreover, all participants in the study were US residents. Consequently, it is possible that our results may not generalize to adolescents from other countries, or effects may differ based on schooling situation or race. In addition, because we required participants to complete the baseline assessment before qualifying for the study, our sample may have included adolescents who were particularly motivated, conscientious, etc. Although this helped to reduce the risk of attrition, these participants may have engaged with the intervention differently than we would see in another, less motivated, sample of adolescents.

Another important consideration is that because we advertised to caregivers and obtained parental consent, our participants may have also differed from the general population in terms of attachment style, quality of relationship with their caregiver, parenting styles, or even the caregiver’s own mental health; all of which could impact the extent to which the adolescent responds to and benefits from the program [74,75]. Research suggests that caregiver perceptions of an adolescent’s mental health as well as their attitudes toward mental health care are more predictive of treatment-seeking among adolescents than the adolescents’ own attitudes;, consequently, caregivers may act as the “gatekeepers” to mental health services [76]. Therefore, an important step to improving access to mental health support among adolescents may be to identify opportunities to reach those whose caregivers are less supportive. In future research, obtaining a waiver of parental consent may permit accessing those adolescents who are personally interested in DMHIs, but whose caregivers may be less interested or supportive.

Finally, previous research on DMHIs for adolescents is also criticized for a lack of follow-up assessments [77-79]. However, some interventions, including school-based stress management interventions, may have stronger effects over time than immediately after intervention [27], emphasizing the importance of tracking effects longitudinally. Although this study followed participants for 12 weeks, providing some insight into the longitudinal effects of the intervention, future research should include longer-term follow-up to better ascertain whether effects diminish or strengthen over time. In particular, because rumination can stem from both perceived stress [34] and loneliness [80], it is plausible that there could be continued improvements in rumination resulting from the reductions in perceived stress and loneliness. Similarly, additional research assessing the long-term implications of Happify for Teens on other mental or physical health outcomes that are predicted by stress, rumination, and loneliness is needed.

### Conclusions

Research suggests that adolescents are experiencing increasing levels of stress, particularly while in school, but few engage in stress management activities [15,16]. Although several stress management interventions have been developed for adolescents, many of these are school-based interventions, and the evidence supporting their effectiveness is mixed [27-29]. Digital interventions may offer more cost-effective and scalable options for addressing important transdiagnostic risk factors in adolescence to promote positive mental health and prevent future dysfunction; however, DMHIs designed specifically for adolescents are rare, particularly those focused on wellness. This study provides promising evidence for the use of a DMHI for addressing perceived stress, brooding, and loneliness in adolescence.

## Appendix

Multimedia Appendix 1 CONSORT-eHEALTH checklist (V 1.6.1).

## Abbreviations

CBT: cognitive behavioral therapy
DMHI: digital mental health intervention
MLM: multilevel model
PROMIS: Patient-Reported Outcomes Measurement Information System
RCT: randomized controlled trial

## References

[1] Kessler RC, Berglund P, Demler O, Jin R, Merikangas KR, Walters EE (2005). Lifetime prevalence and age-of-onset distributions of DSM-IV disorders in the National Comorbidity Survey Replication. Arch Gen Psychiatry.

[2] Kessler RC, Angermeyer M, Anthony JC, De Graaf R, Demyttenaere K, Gasquet I, De Girolamo G, Gluzman S, Gureje O, Haro JM, Kawakami N, Karam A, Levinson D, Mora MEM, Browne MAO, Posada-Villa J, Stein DJ, Tsang CHA, Aguilar-Gaxiola S, Alonso J, Lee S, Heeringa S, Pennell BE, Berglund P, Gruber MJ, Petukhova M, Chatterji S, Ustün TB (2007). Lifetime prevalence and age-of-onset distributions of mental disorders in the World Health Organization's World Mental Health Survey Initiative. World Psychiatry.

[3] Mental health atlas 2014. World Health Organization.

[4] Twenge JM, Cooper AB, Joiner TE, Duffy ME, Binau SG (2019). Age, period, and cohort trends in mood disorder indicators and suicide-related outcomes in a nationally representative dataset, 2005-2017. J Abnorm Psychol.

[5] Olfson M, Blanco C, Wang S, Laje G, Correll CU (2014). National trends in the mental health care of children, adolescents, and adults by office-based physicians. JAMA Psychiatry.

[6] Merikangas KR, He JP, Burstein M, Swendsen J, Avenevoli S, Case B, Georgiades K, Heaton L, Swanson S, Olfson M (2011). Service utilization for lifetime mental disorders in U.S. adolescents: results of the National Comorbidity Survey-Adolescent Supplement (NCS-A). J Am Acad Child Adolesc Psychiatry.

[7] Whitney DG, Peterson MD (2019). US national and state-level prevalence of mental health disorders and disparities of mental health care use in children. JAMA Pediatr.

[8] Gulliver A, Griffiths KM, Christensen H (2010). Perceived barriers and facilitators to mental health help-seeking in young people: a systematic review. BMC Psychiatry.

[9] Olfson M, Druss BG, Marcus SC (2015). Trends in mental health care among children and adolescents. N Engl J Med.

[10] Thomas CR, Holzer CE (2006). The continuing shortage of child and adolescent psychiatrists. J Am Acad Child Adolesc Psychiatry.

[11] Kazdin AE (1993). Adolescent mental health. Prevention and treatment programs. Am Psychol.

[12] Romeo RD (2013). The teenage brain: the stress response and the adolescent brain. Curr Dir Psychol Sci.

[13] Suo L, Zhao L, Si J, Liu J, Zhu W, Chai B, Zhang Y, Feng J, Ding Z, Luo Y, Shi H, Shi J, Lu L (2013). Predictable chronic mild stress in adolescence increases resilience in adulthood. Neuropsychopharmacology.

[14] Østerås B, Sigmundsson H, Haga M (2015). Perceived stress and musculoskeletal pain are prevalent and significantly associated in adolescents: an epidemiological cross-sectional study. BMC Public Health.

[15] (2014). Stress in America™: are teens adopting adults' stress habits?. American Psychological Association.

[16] (2020). Stress in America 2020™: a national mental health crisis. American Psychological Association.

[17] Burger K, Samuel R (2017). The role of perceived stress and self-efficacy in young people's life satisfaction: a longitudinal study. J Youth Adolesc.

[18] DuBois DL, Felner RD, Brand S, Adan AM, Evans EG (1992). A prospective study of life stress, social support, and adaptation in early adolescence. Child Dev.

[19] Kaplan DS, Liu RX, Kaplan HB (2005). School related stress in early adolescence and academic performance three years later: the conditional influence of self expectations. Soc Psychol Educ.

[20] Wills TA, Sandy JM, Yaeger AM (2002). Stress and smoking in adolescence: a test of directional hypotheses. Health Psychol.

[21] Nguyen-Rodriguez ST, Chou CP, Unger JB, Spruijt-Metz D (2008). BMI as a moderator of perceived stress and emotional eating in adolescents. Eat Behav.

[22] De Vriendt T, Clays E, Huybrechts I, De Bourdeaudhuij I, Moreno LA, Patterson E, Molnár D, Mesana MI, Beghin L, Widhalm K, Manios Y, De Henauw S (2012). European adolescents' level of perceived stress is inversely related to their diet quality: the healthy lifestyle in Europe by nutrition in adolescence study. Br J Nutr.

[23] Wiklund M, Malmgren-Olsson EB, Ohman A, Bergström E, Fjellman-Wiklund A (2012). Subjective health complaints in older adolescents are related to perceived stress, anxiety and gender—a cross-sectional school study in Northern Sweden. BMC Public Health.

[24] Wang S, Zhao Y, Zhang L, Wang X, Wang X, Cheng B, Luo K, Gong Q (2019). Stress and the brain: perceived stress mediates the impact of the superior frontal gyrus spontaneous activity on depressive symptoms in late adolescence. Hum Brain Mapp.

[25] Lathren C, Bluth K, Park J (2019). Adolescent self-compassion moderates the relationship between perceived stress and internalizing symptoms. Pers Individ Dif.

[26] Carter JS, Garber J (2011). Predictors of the first onset of a major depressive episode and changes in depressive symptoms across adolescence: stress and negative cognitions. J Abnorm Psychol.

[27] van Loon AWG, Creemers HE, Beumer WY, Okorn A, Vogelaar S, Saab N, Miers AC, Westenberg PM, Asscher JJ (2020). Can schools reduce adolescent psychological stress? A multilevel meta-analysis of the effectiveness of school-based intervention programs. J Youth Adolesc.

[28] Feiss R, Dolinger SB, Merritt M, Reiche E, Martin K, Yanes JA, Thomas CM, Pangelinan M (2019). A systematic review and meta-analysis of school-based stress, anxiety, and depression prevention programs for adolescents. J Youth Adolesc.

[29] Rew L, Johnson K, Young C (2014). A systematic review of interventions to reduce stress in adolescence. Issues Ment Health Nurs.

[30] Lindahl M, Archer T (2013). Depressive expression and anti-depressive protection in adolescence: stress, positive affect, motivation and self-efficacy. Psychology.

[31] Dozois DJA, Seeds PM, Collins KA (2009). Transdiagnostic approaches to the prevention of depression and anxiety. J Cogn Psychother.

[32] Kuhlman KR, Chiang JJ, Bower JE, Irwin MR, Cole SW, Dahl RE, Almeida DM, Fuligni AJ (2020). Persistent low positive affect and sleep disturbance across adolescence moderate link between stress and depressive symptoms in early adulthood. J Abnorm Child Psychol.

[33] Smith JM, Alloy LB (2009). A roadmap to rumination: a review of the definition, assessment, and conceptualization of this multifaceted construct. Clin Psychol Rev.

[34] Michl LC, McLaughlin KA, Shepherd K, Nolen-Hoeksema S (2013). Rumination as a mechanism linking stressful life events to symptoms of depression and anxiety: longitudinal evidence in early adolescents and adults. J Abnorm Psychol.

[35] Skitch SA, Abela JRZ (2008). Rumination in response to stress as a common vulnerability factor to depression and substance misuse in adolescence. J Abnorm Child Psychol.

[36] Marks ADG, Sobanski DJ, Hine DW (2010). Do dispositional rumination and/or mindfulness moderate the relationship between life hassles and psychological dysfunction in adolescents?. Aust N Z J Psychiatry.

[37] McLaughlin KA, Nolen-Hoeksema S (2012). Interpersonal stress generation as a mechanism linking rumination to internalizing symptoms in early adolescents. J Clin Child Adolesc Psychol.

[38] Beck AT (1979). Cognitive Therapy of Depression.

[39] Praissman S (2008). Mindfulness-based stress reduction: a literature review and clinician's guide. J Am Acad Nurse Pract.

[40] Duckworth AL, Steen TA, Seligman ME (2005). Positive psychology in clinical practice. Annu Rev Clin Psychol.

[41] Seligman MEP, Steen TA, Park N, Peterson C (2005). Positive psychology progress: empirical validation of interventions. Am Psychol.

[42] Edwards M, Adams EM, Waldo M, Hadfield OD, Biegel GM (2014). Effects of a mindfulness group on Latino adolescent students: examining levels of perceived stress, mindfulness, self-compassion, and psychological symptoms. J Spec Group Work.

[43] Hamdan-Mansour AM, Puskar K, Bandak AG (2009). Effectiveness of cognitive-behavioral therapy on depressive symptomatology, stress and coping strategies among Jordanian university students. Issues Ment Health Nurs.

[44] Brinkborg H, Michanek J, Hesser H, Berglund G (2011). Acceptance and commitment therapy for the treatment of stress among social workers: a randomized controlled trial. Behav Res Ther.

[45] Wersebe H, Lieb R, Meyer AH, Hofer P, Gloster AT (2018). The link between stress, well-being, and psychological flexibility during an Acceptance and Commitment Therapy self-help intervention. Int J Clin Health Psychol.

[46] Carpenter J, Crutchley P, Zilca RD, Schwartz HA, Smith LK, Cobb AM, Parks AC (2016). Seeing the "big" picture: big data methods for exploring relationships between usage, language, and outcome in internet intervention data. J Med Internet Res.

[47] Carpenter J, Crutchley P, Zilca RD, Schwartz HA, Smith LK, Cobb AM, Parks AC (2017). Correction: seeing the "big" picture: big data methods for exploring relationships between usage, language, and outcome in internet intervention data. J Med Internet Res.

[48] Parks AC, Williams AL, Kackloudis GM, Stafford JL, Boucher EM, Honomichl RD (2020). The effects of a digital well-being intervention on patients with chronic conditions: observational study. J Med Internet Res.

[49] Parks AC, Williams AL, Tugade MM, Hokes KE, Honomichl RD, Zilca RD (2018). Testing a scalable web and smartphone based intervention to improve depression, anxiety, and resilience: a randomized controlled trial. Int J Wellbeing.

[50] Williams AL, Parks AC, Cormier G, Stafford J, Whillans A (2018). Improving resilience among employees high in depression, anxiety, and workplace distress. Int J Manag Res.

[51] Boucher EM, Ward HE, Stafford JL, Parks AC (2021). Effects of a digital mental health program on perceived stress in adolescents aged 13-17 years: protocol for a randomized controlled trial. JMIR Res Protoc.

[52] Cohen S, Kamarck T, Mermelstein R (1983). A global measure of perceived stress. J Health Soc Behav.

[53] Treynor W, Gonzalez R, Nolen-Hoeksema S (2003). Rumination reconsidered: a psychometric analysis. Cogn Ther Res.

[54] Baumel A, Edan S, Kane JM (2019). Is there a trial bias impacting user engagement with unguided e-mental health interventions? A systematic comparison of published reports and real-world usage of the same programs. Transl Behav Med.

[55] Forrest CB, Meltzer LJ, Marcus CL, de la Motte A, Kratchman A, Buysse DJ, Pilkonis PA, Becker BD, Bevans KB (2018). Development and validation of the PROMIS Pediatric Sleep Disturbance and Sleep-Related Impairment item banks. Sleep.

[56] Roberts RE, Lewinsohn PM, Seeley JR (1993). A brief measure of loneliness suitable for use with adolescents. Psychol Rep.

[57] Scheier MF, Carver CS, Bridges MW (1994). Distinguishing optimism from neuroticism (and trait anxiety, self-mastery, and self-esteem): a reevaluation of the Life Orientation Test. J Pers Soc Psychol.

[58] Bates D, Mächler M, Bolker B, Walker S (2015). Fitting linear mixed-effects models using lme4. J Stat Softw.

[59] Wood D, Harms PD, Lowman GH, DeSimone JA (2017). Response speed and response consistency as mutually validating indicators of data quality in online samples. Soc Psychol Pers Sci.

[60] Eysenbach G (2005). The law of attrition. J Med Internet Res.

[61] Boucher E, Honomichl R, Ward H, Powell T, Stoeckl SE, Parks A (2022). The effects of a digital well-being intervention on older adults: retrospective analysis of real-world user data. JMIR Aging.

[62] Montgomery RM, Boucher EM, Honomichl RD, Powell TA, Guyton SL, Bernecker SL, Stoeckl SE, Parks AC (2021). The effects of a digital mental health intervention in adults with cardiovascular disease risk factors: analysis of real-world user data. JMIR Cardio.

[63] Lehtimaki S, Martic J, Wahl B, Foster KT, Schwalbe N (2021). Evidence on digital mental health interventions for adolescents and young people: systematic overview. JMIR Ment Health.

[64] Boucher EM, McNaughton EC, Harake N, Stafford JL, Parks AC (2021). The impact of a digital intervention (Happify) on loneliness during COVID-19: qualitative focus group. JMIR Ment Health.

[65] Hoeppner SS, Millstein RA, Siegel KR, Carlon HA, Harnedy LE, Chung WJ, Huffman JC, Hoeppner BB (2022). A secondary analysis examining the performance of the State Optimism Measure (SOM) compared to the Life Orientation Test-Revised (LOT-R) in measuring optimism over time. Psychol Health.

[66] Hicks TB, Shahidullah JD, Carlson JS, Palejwala MH (2014). Nationally Certified School Psychologists' use and reported barriers to using evidence-based interventions in schools: the influence of graduate program training and education. Sch Psychol Q.

[67] Gronholm PC, Nye E, Michelson D (2018). Stigma related to targeted school-based mental health interventions: a systematic review of qualitative evidence. J Affect Disord.

[68] (2018). Teens, social media and technology 2018. Pew Research Center.

[69] Martínez-Pérez B, de la Torre-Díez I, López-Coronado M (2013). Mobile health applications for the most prevalent conditions by the World Health Organization: review and analysis. J Med Internet Res.

[70] Grist R, Porter J, Stallard P (2017). Mental health mobile apps for preadolescents and adolescents: a systematic review. J Med Internet Res.

[71] Kulikov V, Padmanabhan A, Peake E, Lake J (2022). 1.29 An evaluation of the effectiveness of a CBT-based digital therapeutic in reducing adolescent depressive symptoms. J Am Acad Child Adolesc Psychiatry.

[72] Torous J, Firth J (2016). The digital placebo effect: mobile mental health meets clinical psychiatry. Lancet Psychiatry.

[73] Goldberg SB, Lam SU, Simonsson O, Torous J, Sun S (2022). Mobile phone-based interventions for mental health: a systematic meta-review of 14 meta-analyses of randomized controlled trials. PLOS Digit Health.

[74] Melchior M, van der Waerden J (2016). Parental influences on children's mental health: the bad and the good sides of it. Eur Child Adolesc Psychiatry.

[75] DeWalt DA, Hink A (2009). Health literacy and child health outcomes: a systematic review of the literature. Pediatrics.

[76] Tsang YT, Franklin M, Sala-Hamrick K, Kohlberger B, Simon VA, Partridge T, Barnett D (2020). Caregivers as gatekeepers: professional mental health service use among urban minority adolescents. Am J Orthopsychiatry.

[77] Richardson T, Stallard P, Velleman S (2010). Computerised cognitive behavioural therapy for the prevention and treatment of depression and anxiety in children and adolescents: a systematic review. Clin Child Fam Psychol Rev.

[78] Li J, Theng YL, Foo S (2014). Game-based digital interventions for depression therapy: a systematic review and meta-analysis. Cyberpsychol Behav Soc Netw.

[79] Hollis C, Falconer CJ, Martin JL, Whittington C, Stockton S, Glazebrook C, Davies EB (2017). Annual research review: digital health interventions for children and young people with mental health problems—a systematic and meta-review. J Child Psychol Psychiatry.

[80] Vanhalst J, Luyckx K, Van Petegem S, Soenens B (2018). The detrimental effects of adolescents' chronic loneliness on motivation and emotion regulation in social situations. J Youth Adolesc.

