# Supplementary material for: Effects of a Digital Mental Health Intervention on Perceived Stress and Rumination in Adolescents Aged 13 to 17 Years: Randomized Controlled Trial
Source: J Med Internet Res. 2024 Mar 29;26:e54282. doi: 10.2196/54282 (PMC11015368; doi:10.2196/54282)
Supplement: Multimedia Appendix 1 [file jmir_v26i1e54282_app1.pdf]

CONSORT-EHEALTH (V 1.6.1) - Submission/Publication Form

The CONSORT-EHEALTH checklist is intended for authors of randomized trials evaluating web-based and Internet-based applications/interventions, including mobile interventions, electronic games (incl multiplayer games), social media, certain telehealth applications, and other interactive and/or networked electronic applications. Some of the items (e.g. all subitems under item 5 - description of the intervention) may also be applicable for other study designs.

The goal of the CONSORT EHEALTH checklist and guideline is to be
a) a guide for reporting for authors of RCTs,
b) to form a basis for appraisal of an ehealth trial (in terms of validity)

CONSORT-EHEALTH items/subitems are MANDATORY reporting items for studies published in the Journal of Medical Internet Research and other journals / scientific societies endorsing the checklist.

Items numbered 1., 2., 3., 4a., 4b etc are original CONSORT or CONSORT-NPT (non-pharmacologic treatment) items.
Items with Roman numerals (i., ii, iii, iv etc.) are CONSORT-EHEALTH extensions/clarifications.

As the CONSORT-EHEALTH checklist is still considered in a formative stage, we would ask that you also RATE ON A SCALE OF 1-5 how important/useful you feel each item is FOR THE PURPOSE OF THE CHECKLIST and reporting guideline (optional).

Mandatory reporting items are marked with a red *.
In the textboxes, either copy & paste the relevant sections from your manuscript into this form - please include any quotes from your manuscript in QUOTATION MARKS,
or answer directly by providing additional information not in the manuscript, or elaborating on why the item was not relevant for this study.

YOUR ANSWERS WILL BE PUBLISHED AS A SUPPLEMENTARY FILE TO YOUR PUBLICATION IN JMIR AND ARE CONSIDERED PART OF YOUR PUBLICATION (IF ACCEPTED).
Please fill in these questions diligently. Information will not be copyedited, so please use proper spelling and grammar, use correct capitalization, and avoid abbreviations.

DO NOT FORGET TO SAVE AS PDF _AND_ CLICK THE SUBMIT BUTTON SO YOUR ANSWERS ARE IN OUR DATABASE !!!

Citation Suggestion (if you append the pdf as Appendix we suggest to cite this paper in the caption):
Eysenbach G, CONSORT-EHEALTH Group

CONSORT-EHEALTH: Improving and Standardizing Evaluation Reports of Web-based and Mobile Health Interventions
J Med Internet Res 2011;13(4):e126
URL: http://www.jmir.org/2011/4/e126/
doi: 10.2196/jmir.1923
PMID: 22209829

eliane@twill.health Switch account

Not shared

Draft saved

* Indicates required question

Your name *

First Last

Eliane Boucher

Primary Affiliation (short), City, Country *

University of Toronto, Toronto, Canada

Twill

Your e-mail address *

abc@gmail.com

eliane@twill.health

Title of your manuscript *

Provide the (draft) title of your manuscript.

Effects of a Digital Mental Health Intervention on Perceived Stress and Rumination in Adolescents Aged 13 to 17: A Randomized Controlled Trial

Name of your App/Software/Intervention *

If there is a short and a long/alternate name, write the short name first and add the long name in brackets.

Happify for Teens

Evaluated Version (if any)

e.g. "V1", "Release 2017-03-01", "Version 2.0.27913"

Your answer

Language(s) *

What language is the intervention/app in? If multiple languages are available, separate by comma (e.g. "English, French")

English

URL of your Intervention Website or App

e.g. a direct link to the mobile app on app in appstore (itunes, Google Play), or URL of the website. If the intervention is a DVD or hardware, you can also link to an Amazon page.

https://www.happify.com/

URL of an image/screenshot (optional)

Your answer

Accessibility *

Can an enduser access the intervention presently?

- ( ) access is free and open
- (x) access only for special usergroups, not open
- ( ) access is open to everyone, but requires payment/subscription/in-app purchases
- ( ) app/intervention no longer accessible
- ( ) Other:

Primary Medical Indication/Disease/Condition *

e.g. "Stress", "Diabetes", or define the target group in brackets after the condition, e.g. "Autism (Parents of children with)", "Alzheimers (Informal Caregivers of)"

Stress (Adolescents)

Primary Outcomes measured in trial *

comma-separated list of primary outcomes reported in the trial

Perceived Stress

Secondary/other outcomes

Are there any other outcomes the intervention is expected to affect?

Rumination/Brooding, Loneliness

Recommended "Dose" *

What do the instructions for users say on how often the app should be used?

- ( ) Approximately Daily
- (x) Approximately Weekly
- ( ) Approximately Monthly
- ( ) Approximately Yearly
- ( ) "as needed"
- ( ) Other:

Approx. Percentage of Users (starters) still using the app as recommended after 3 months *

- (x) unknown / not evaluated
- ( ) 0-10%
- ( ) 11-20%
- ( ) 21-30%
- ( ) 31-40%
- ( ) 41-50%
- ( ) 51-60%
- ( ) 61-70%
- ( ) 71%-80%
- ( ) 81-90%
- ( ) 91-100%
- ( ) Other:

Overall, was the app/intervention effective? *

- (selected) yes: all primary outcomes were significantly better in intervention group vs control
- partly: SOME primary outcomes were significantly better in intervention group vs control
- no statistically significant difference between control and intervention
- potentially harmful: control was significantly better than intervention in one or more outcomes
- inconclusive: more research is needed
- Other:

Article Preparation Status/Stage *

At which stage in your article preparation are you currently (at the time you fill in this form)

- not submitted yet - in early draft status
- not submitted yet - in late draft status, just before submission
- (selected) submitted to a journal but not reviewed yet
- submitted to a journal and after receiving initial reviewer comments
- submitted to a journal and accepted, but not published yet
- published
- Other:

Journal *

If you already know where you will submit this paper (or if it is already submitted), please provide the journal name (if it is not JMIR, provide the journal name under "other")

- ○ not submitted yet / unclear where I will submit this
- ◉ Journal of Medical Internet Research (JMIR)
- ○ JMIR mHealth and UHealth
- ○ JMIR Serious Games
- ○ JMIR Mental Health
- ○ JMIR Public Health
- ○ JMIR Formative Research
- ○ Other JMIR sister journal
- ○ Other:

Is this a full powered effectiveness trial or a pilot/feasibility trial? *

- ○ Pilot/feasibility
- ◉ Fully powered

Manuscript tracking number *

If this is a JMIR submission, please provide the manuscript tracking number under "other" (The ms tracking number can be found in the submission acknowledgement email, or when you login as author in JMIR. If the paper is already published in JMIR, then the ms tracking number is the four-digit number at the end of the DOI, to be found at the bottom of each published article in JMIR)

- ○ no ms number (yet) / not (yet) submitted to / published in JMIR
- ◉ Other: 54282

TITLE AND ABSTRACT

1a) TITLE: Identification as a randomized trial in the title

1a) Does your paper address CONSORT item 1a? *

I.e does the title contain the phrase "Randomized Controlled Trial"? (if not, explain the reason under "other")

- (selected) yes
- ( ) Other:

1a-i) Identify the mode of delivery in the title

Identify the mode of delivery. Preferably use “web-based” and/or “mobile” and/or “electronic game” in the title. Avoid ambiguous terms like “online”, “virtual”, “interactive”. Use “Internet-based” only if Intervention includes non-web-based Internet components (e.g. email), use “computer-based” or “electronic” only if offline products are used. Use “virtual” only in the context of “virtual reality” (3-D worlds). Use “online” only in the context of “online support groups”. Complement or substitute product names with broader terms for the class of products (such as “mobile” or “smart phone” instead of “iphone”), especially if the application runs on different platforms.

	1	2	3	4	5	
subitem not at all important	( )	( )	(selected)	( )	( )	essential

Clear selection

Does your paper address subitem 1a-i? *

Copy and paste relevant sections from manuscript title (include quotes in quotation marks "like this" to indicate direct quotes from your manuscript), or elaborate on this item by providing additional information not in the ms, or briefly explain why the item is not applicable/relevant for your study

"Effects of a Digital Mental Health Intervention on Perceived Stress and Rumination in Adolescents Aged 13 to 17" - note that "digital" is used to conform to established terminology in the scientific literature for digital mental health interventions, and because the intervention can be accessed from a smartphone, computer, or tablet.

1a-ii) Non-web-based components or important co-interventions in title

Mention non-web-based components or important co-interventions in title, if any (e.g., “with telephone support”).

	1	2	3	4	5	
subitem not at all important	◉	○	○	○	○	essential

Clear selection

Does your paper address subitem 1a-ii?

Copy and paste relevant sections from manuscript title (include quotes in quotation marks "like this" to indicate direct quotes from your manuscript), or elaborate on this item by providing additional information not in the ms, or briefly explain why the item is not applicable/relevant for your study

There are no non-web-based components or co-interventions.

1a-iii) Primary condition or target group in the title

Mention primary condition or target group in the title, if any (e.g., “for children with Type I Diabetes”) Example: A Web-based and Mobile Intervention with Telephone Support for Children with Type I Diabetes: Randomized Controlled Trial

	1	2	3	4	5	
subitem not at all important	○	○	○	●	○	essential

Clear selection

Does your paper address subitem 1a-iii? *

Copy and paste relevant sections from manuscript title (include quotes in quotation marks "like this" to indicate direct quotes from your manuscript), or elaborate on this item by providing additional information not in the ms, or briefly explain why the item is not applicable/relevant for your study

Target group is described: "in Adolescents Aged 13 to 17".

1b) ABSTRACT: Structured summary of trial design, methods, results, and conclusions

NPT extension: Description of experimental treatment, comparator, care providers, centers, and blinding status.

1b-i) Key features/functionalities/components of the intervention and comparator in the METHODS section of the ABSTRACT

Mention key features/functionalities/components of the intervention and comparator in the abstract. If possible, also mention theories and principles used for designing the site. Keep in mind the needs of systematic reviewers and indexers by including important synonyms. (Note: Only report in the abstract what the main paper is reporting. If this information is missing from the main body of text, consider adding it)

	1	2	3	4	5	
subitem not at all important	○	○	○	◉	○	essential

Clear selection

Does your paper address subitem 1b-i? *

Copy and paste relevant sections from the manuscript abstract (include quotes in quotation marks "like this" to indicate direct quotes from your manuscript), or elaborate on this item by providing additional information not in the ms, or briefly explain why the item is not applicable/relevant for your study

"Participants were randomly assigned to engage with a self-guided DMHI for adolescents (Happify for Teens) or to a waitlist control. Participants assigned to the intervention group were given access to the program for 12 weeks. Happify for Teens consists of various evidence-based activities drawn from therapeutic modalities such as Cognitive Behavioral Therapy, Positive Psychology and Mindfulness, which are then organized into several programs targeting specific areas of concern (e.g., Stress Buster 101). Participants in the waitlist control received access to this product for 12 weeks upon completing the study."

1b-ii) Level of human involvement in the METHODS section of the ABSTRACT

Clarify the level of human involvement in the abstract, e.g., use phrases like “fully automated” vs. “therapist/nurse/care provider/physician-assisted” (mention number and expertise of providers involved, if any). (Note: Only report in the abstract what the main paper is reporting. If this information is missing from the main body of text, consider adding it)

	1	2	3	4	5	
subitem not at all important	○	○	○	◉	○	essential

Clear selection

Does your paper address subitem 1b-ii?

Copy and paste relevant sections from the manuscript abstract (include quotes in quotation marks "like this" to indicate direct quotes from your manuscript), or elaborate on this item by providing additional information not in the ms, or briefly explain why the item is not applicable/relevant for your study

The intervention is described as self-guided: "Participants were randomly assigned to engage with a self-guided DMHI for adolescents (Happify for Teens) or to a waitlist control"

1b-iii) Open vs. closed, web-based (self-assessment) vs. face-to-face assessments in the METHODS section of the ABSTRACT

Mention how participants were recruited (online vs. offline), e.g., from an open access website or from a clinic or a closed online user group (closed usergroup trial), and clarify if this was a purely web-based trial, or there were face-to-face components (as part of the intervention or for assessment). Clearly say if outcomes were self-assessed through questionnaires (as common in web-based trials). Note: In traditional offline trials, an open trial (open-label trial) is a type of clinical trial in which both the researchers and participants know which treatment is being administered. To avoid confusion, use “blinded” or “unblinded” to indicated the level of blinding instead of “open”, as “open” in web-based trials usually refers to “open access” (i.e. participants can self-enrol). (Note: Only report in the abstract what the main paper is reporting. If this information is missing from the main body of text, consider adding it)

	1	2	3	4	5	
subitem not at all important	○	○	○	●	○	essential

Clear selection

Does your paper address subitem 1b-iii?

Copy and paste relevant sections from the manuscript abstract (include quotes in quotation marks "like this" to indicate direct quotes from your manuscript), or elaborate on this item by providing additional information not in the ms, or briefly explain why the item is not applicable/relevant for your study

The trial is described as "decentralized" to clarify that recruitment and assessment were completed online. "Participants in both groups completed measures of perceived stress, brooding, optimism, sleep disturbance, and loneliness at baseline, 4 weeks, 8 weeks, and 12 weeks." describes the outcomes were self-reported. Because the study has a waitlist control, it is implied that the study is unblinded.

1b-iv) RESULTS section in abstract must contain use data

Report number of participants enrolled/assessed in each group, the use/uptake of the intervention (e.g., attrition/adherence metrics, use over time, number of logins etc.), in addition to primary/secondary outcomes. (Note: Only report in the abstract what the main paper is reporting. If this information is missing from the main body of text, consider adding it)

	1	2	3	4	5	
subitem not at all important	○	○	●	○	○	essential

Clear selection

Does your paper address subitem 1b-iv?

Copy and paste relevant sections from the manuscript abstract (include quotes in quotation marks "like this" to indicate direct quotes from your manuscript), or elaborate on this item by providing additional information not in the ms, or briefly explain why the item is not applicable/relevant for your study

Number of participants in each group is reported: "Of the 303 participants included in data analyses, 132 were assigned to the intervention and 171 to the waitlist." Usage is not reported in the manuscript as the focus is on differences across the two conditions and there is no version of usage for the waitlist control.

1b-v) CONCLUSIONS/DISCUSSION in abstract for negative trials

Conclusions/Discussions in abstract for negative trials: Discuss the primary outcome - if the trial is negative (primary outcome not changed), and the intervention was not used, discuss whether negative results are attributable to lack of uptake and discuss reasons. (Note: Only report in the abstract what the main paper is reporting. If this information is missing from the main body of text, consider adding it)

	1	2	3	4	5	
subitem not at all important	●	○	○	○	○	essential

Clear selection

Does your paper address subitem 1b-v?

Copy and paste relevant sections from the manuscript abstract (include quotes in quotation marks "like this" to indicate direct quotes from your manuscript), or elaborate on this item by providing additional information not in the ms, or briefly explain why the item is not applicable/relevant for your study

The trial was successful; these effects are described in the abstract, as are the two non-significant findings for exploratory outcomes: "There were significantly greater improvements in the intervention condition for perceived stress (intervention 95% CI: -1.82 – -1.19, p<.001; control 95% CI: -0.44 – 0.26, p=.607), brooding (intervention: 95% CI: -1.00 – -0.68, p<.001; waitlist: 95% CI: -0.47 – -0.12; p=.001), and loneliness (intervention: 95% CI: -1.2 – -0.73, p<.001; control: 95% CI: -0.64 – -0.12, p=.005) over the 12-week study period. Changes in optimism and sleep disturbance were not significantly different across groups (ps≥.096)."

INTRODUCTION

2a) In INTRODUCTION: Scientific background and explanation of rationale

2a-i) Problem and the type of system/solution

Describe the problem and the type of system/solution that is object of the study: intended as stand-alone intervention vs. incorporated in broader health care program? Intended for a particular patient population? Goals of the intervention, e.g., being more cost-effective to other interventions, replace or complement other solutions? (Note: Details about the intervention are provided in "Methods" under 5)

	1	2	3	4	5	
subitem not at all important	○	○	○	◉	○	essential

Clear selection

Does your paper address subitem 2a-i? *

Copy and paste relevant sections from the manuscript (include quotes in quotation marks "like this" to indicate direct quotes from your manuscript), or elaborate on this item by providing additional information not in the ms, or briefly explain why the item is not applicable/relevant for your study

The solution is described as focusing on a gap in solutions to support adolescents with subclinical mental health concerns: "Interventions focused more on promoting flourishing and preventing future dysfunction and impairment may help to reduce the burden on these services by offering alternative services to adolescents with little or no mental health impairment [11]." and the program/study is described in more detail under the Current Study Objectives: "The aim of the current study was to test the impact of a digital mental health intervention (DMHI) designed for adolescents called Happify for Teens. This program has been adapted for 13- to 17-years olds from another DMHI developed for adults (Happify). Happify for Teens consists of gamified versions of evidence-based activities adapted from various therapeutic approaches including CBT [38], mindfulness-based stress reduction (MBSR) [39], and positive psychology (PP) [40,41]. Consequently, these programs integrate a variety of recommended approaches to addressing negative cognitions [31] that also help manage stress [42-45]."

2a-ii) Scientific background, rationale: What is known about the (type of) system

Scientific background, rationale: What is known about the (type of) system that is the object of the study (be sure to discuss the use of similar systems for other conditions/diagnoses, if appropiate), motivation for the study, i.e. what are the reasons for and what is the context for this specific study, from which stakeholder viewpoint is the study performed, potential impact of findings [2]. Briefly justify the choice of the comparator.

	1	2	3	4	5	
subitem not at all important	○	○	○	◉	○	essential

Clear selection

Does your paper address subitem 2a-ii? *

Copy and paste relevant sections from the manuscript (include quotes in quotation marks "like this" to indicate direct quotes from your manuscript), or elaborate on this item by providing additional information not in the ms, or briefly explain why the item is not applicable/relevant for your study

"Adolescence is a developmental stage where individuals may be particularly sensitive to stress due to shifts in hypothalamic-pituitary-adrenal (HPA) axis reactivity, leading to more intense hormonal responses to stressors, particularly in older adolescents [12]. Over the last decade, researchers have become increasingly interested in adolescent stress as an important transdiagnostic risk factor. Although animal models suggest that predictable, chronic mild stress levels in adolescence can actually increase resilience in adulthood [13], research suggests many adolescents are coping with more severe stress levels. In one study, 22% of adolescents aged 15 to 17 reported moderate to severe levels of perceived stress [14]. In addition, national surveys conducted by the American Psychological Association (APA) have found that adolescents report higher levels of stress than most adults, particularly during the school year [15,16].

Such high levels of stress have been linked to lower life satisfaction [17], poorer academic performance [18,19], cigarette smoking [20], emotional eating [21], poorer diet [22], more frequent subjective health complaints (e.g., headaches, fatigue, sleep difficulties) [23], as well as internalizing symptoms including depression [24,25] and anxiety [25]. Interpersonal stress, in particular, predicts the onset of a first major depressive episode among adolescents [26]. Thus addressing stress in adolescents is critical to avoiding more long-term problems.

However, 25% of adolescents self-report not doing enough to manage their stress [16]. Approximately 55% of adolescents report setting aside time for stress management "a few times a month or less", 13% report never setting aside time for stress management, and just 5% report having seen a mental health professional about their stress [15]. Based on these data, the APA noted the need for opportunities to help adolescents address and cope with their stress in order "to break this unhealthy legacy of stress in America" [15].

Although there has been an increase in interventions targeting adolescent stress [27], evidence-based interventions for stress are much less common than those for anxiety and depression [28]. Furthermore, most interventions for stress are school-based, and the evidence for these interventions is mixed. In one systematic review of stress management interventions, only 58% of the reviewed studies found significant improvements in physiological indicators of stress (e.g., blood pressure) or self-reported stress [29]. More recent meta-analyses of school-based programs further suggest that these programs may only be effective for school rather than social stress [27]. Given these findings, we need more research exploring the effectiveness of stress-management programs with adolescents, particularly those that could be delivered outside schools."

2b) In INTRODUCTION: Specific objectives or hypotheses

Does your paper address CONSORT subitem 2b? *

Copy and paste relevant sections from the manuscript (include quotes in quotation marks "like this" to indicate direct quotes from your manuscript), or elaborate on this item by providing additional information not in the ms, or briefly explain why the item is not applicable/relevant for your study

"The aim of the current study was to test the impact of a digital mental health intervention (DMHI) designed for adolescents called Happify for Teens. This program has been adapted for 13- to 17-years olds from another DMHI developed for adults (Happify). Happify for Teens consists of gamified versions of evidence-based activities adapted from various therapeutic approaches including CBT [38], mindfulness-based stress reduction (MBSR) [39], and positive psychology (PP) [40,41]. Consequently, these programs integrate a variety of recommended approaches to addressing negative cognitions [31] that also help manage stress [42-45].

Observational studies of real-world Happify users have shown significant improvements in subjective well-being and anxiety among adults after 6-8 weeks of usage, with greater gains among users who completed more activities [46-48]. Randomized controlled trials (RCTs) with adult participants have similarly shown improvements in depressive symptoms, anxiety, and resilience among those who completed at least two activities per week relative to a control group [49,50]. However, Happify for Teens has yet to be tested empirically. Therefore, in the current study, we recruited adolescents aged 13 to 17 who reported elevated stress and rumination. Participants were randomly assigned to have access to Happify for Teens for 12 weeks, or to a corresponding waitlist control group. We compared changes in perceived stress after 4 weeks, 8 weeks, and 12 weeks across the two groups. We also examined changes in brooding (the maladaptive component of rumination) as a secondary outcome, and in sleep disturbance, loneliness, and optimism as exploratory outcomes."

METHODS

3a) Description of trial design (such as parallel, factorial) including allocation ratio

Does your paper address CONSORT subitem 3a? *

Copy and paste relevant sections from the manuscript (include quotes in quotation marks "like this" to indicate direct quotes from your manuscript), or elaborate on this item by providing additional information not in the ms, or briefly explain why the item is not applicable/relevant for your study

"This was a 12-week, two-arm RCT of adolescents aged 13 to 17 residing in the U.S. with elevated levels of perceived stress and brooding. A study protocol was previously published [51] and the trial was registered with clinicaltrials.gov (NCT04567888). Study procedures were reviewed and approved by IntegReview, an independent IRB, which was acquired by Advarra after the study was launched."

3b) Important changes to methods after trial commencement (such as eligibility criteria), with reasons

Does your paper address CONSORT subitem 3b? *

Copy and paste relevant sections from the manuscript (include quotes in quotation marks "like this" to indicate direct quotes from your manuscript), or elaborate on this item by providing additional information not in the ms, or briefly explain why the item is not applicable/relevant for your study

This item is not applicable; there were no changes to study criteria. Other methodological changes made after the published protocol are described in the manuscript including changes to the data analysis plans ("Analyses presented here differ from those in the published protocol [51] because data violated the sphericity assumption for repeated-measures ANOVAs. Additionally, MLMs can manage missing outcome data without imputation, allowing us to retain more participants. Due to power constraints, we focus only on analyses testing changes in primary, secondary, and exploratory outcomes across conditions.") and to sample size ("Note that this sample is smaller than the target sample size reported in the published protocol [51]. As described in that protocol, however, power analyses indicated that 200 participants was sufficient for 80% power to detect a small effect for our primary outcome. Due to the high levels of dropout observed with digital interventions [58], including with adolescents, we assumed a 75% attrition rate and set a target sample size of 800 participants. Because attrition rates were much lower than expected (assessment completion rates ranged from 78.95% to 87.13%), we decided to stop enrollment once 100 participants per condition completed the 12-week assessment to avoid oversampling, resulting in the smaller sample.").

3b-i) Bug fixes, Downtimes, Content Changes

Bug fixes, Downtimes, Content Changes: ehealth systems are often dynamic systems. A description of changes to methods therefore also includes important changes made on the intervention or comparator during the trial (e.g., major bug fixes or changes in the functionality or content) (5-iii) and other “unexpected events” that may have influenced study design such as staff changes, system failures/downtimes, etc. [2].

	1	2	3	4	5	
subitem not at all important	(•)	( )	( )	( )	( )	essential

Clear selection

Does your paper address subitem 3b-i?

Copy and paste relevant sections from the manuscript (include quotes in quotation marks "like this" to indicate direct quotes from your manuscript), or elaborate on this item by providing additional information not in the ms, or briefly explain why the item is not applicable/relevant for your study

No changes were made to the product during the trial.

4a) Eligibility criteria for participants

Does your paper address CONSORT subitem 4a? *

Copy and paste relevant sections from the manuscript (include quotes in quotation marks "like this" to indicate direct quotes from your manuscript), or elaborate on this item by providing additional information not in the ms, or briefly explain why the item is not applicable/relevant for your study

"The study was advertised to parents/guardians via social media, emails to Happify users, within U.S. schools, and using a snowballing recruitment method. Parents/guardians were directed to complete a prescreening questionnaire to report their child's age, country of residence and previous Happify usage, and to provide electronic consent for each eligible child. Adolescents who qualified based on the prescreening questionnaire then were invited to complete a screening questionnaire via email; adolescents were eligible to participate if they reported being 13 to 17 years of age, residing in the U.S., having never used Happify, and reporting elevated stress (Perceived Stress Scale [52] scores > 14) and brooding (Ruminative Responses Scale (RRS) - Short Form - Brooding Subscale scores [53] ≥ 10)."

4a-i) Computer / Internet literacy

Computer / Internet literacy is often an implicit "de facto" eligibility criterion - this should be explicitly clarified.

	1	2	3	4	5	
subitem not at all important	○	◉	○	○	○	essential

Clear selection

Does your paper address subitem 4a-i?

Copy and paste relevant sections from the manuscript (include quotes in quotation marks "like this" to indicate direct quotes from your manuscript), or elaborate on this item by providing additional information not in the ms, or briefly explain why the item is not applicable/relevant for your study

This is not directly addressed in the manuscript; however, the published protocol references that participants must "indicate that they are willing and able to complete the study activities" which includes having access to a smartphone/computer and being willing and able to engage with the digital program.

4a-ii) Open vs. closed, web-based vs. face-to-face assessments:

Open vs. closed, web-based vs. face-to-face assessments: Mention how participants were recruited (online vs. offline), e.g., from an open access website or from a clinic, and clarify if this was a purely web-based trial, or there were face-to-face components (as part of the intervention or for assessment), i.e., to what degree got the study team to know the participant. In online-only trials, clarify if participants were quasi-anonymous and whether having multiple identities was possible or whether technical or logistical measures (e.g., cookies, email confirmation, phone calls) were used to detect/prevent these.

	1	2	3	4	5	
subitem not at all important	○	○	○	◉	○	essential

Clear selection

Does your paper address subitem 4a-ii? *

Copy and paste relevant sections from the manuscript (include quotes in quotation marks "like this" to indicate direct quotes from your manuscript), or elaborate on this item by providing additional information not in the ms, or briefly explain why the item is not applicable/relevant for your study

In several areas in the manuscript, details are provided about the study being fully decentralized and how/when the research team engaged with the participant - either via email or via SMS messaging, depending on the participant's preference.

4a-iii) Information giving during recruitment

Information given during recruitment. Specify how participants were briefed for recruitment and in the informed consent procedures (e.g., publish the informed consent documentation as appendix, see also item X26), as this information may have an effect on user self-selection, user expectation and may also bias results.

	1	2	3	4	5	
subitem not at all important	○	◉	○	○	○	essential

Clear selection

Does your paper address subitem 4a-iii?

Copy and paste relevant sections from the manuscript (include quotes in quotation marks "like this" to indicate direct quotes from your manuscript), or elaborate on this item by providing additional information not in the ms, or briefly explain why the item is not applicable/relevant for your study

Details on recruitment are provided in more detail in the published protocol and therefore are only addressed briefly in the results manuscript.

4b) Settings and locations where the data were collected

Does your paper address CONSORT subitem 4b? *

Copy and paste relevant sections from the manuscript (include quotes in quotation marks "like this" to indicate direct quotes from your manuscript), or elaborate on this item by providing additional information not in the ms, or briefly explain why the item is not applicable/relevant for your study

The manuscript describes the study as decentralized, and describes the sample as US residents only.

4b-i) Report if outcomes were (self-)assessed through online questionnaires

Clearly report if outcomes were (self-)assessed through online questionnaires (as common in web-based trials) or otherwise.

	1	2	3	4	5	
subitem not at all important	○	○	○	●	○	essential

Clear selection

Does your paper address subitem 4b-i? *

Copy and paste relevant sections from the manuscript (include quotes in quotation marks "like this" to indicate direct quotes from your manuscript), or elaborate on this item by providing additional information not in the ms, or briefly explain why the item is not applicable/relevant for your study

"Every four weeks, participants were prompted to complete outcome measures via Qualtrics."

4b-ii) Report how institutional affiliations are displayed

Report how institutional affiliations are displayed to potential participants [on ehealth media], as affiliations with prestigious hospitals or universities may affect volunteer rates, use, and reactions with regards to an intervention.(Not a required item – describe only if this may bias results)

	1	2	3	4	5	
subitem not at all important	◉	○	○	○	○	essential

Clear selection

Does your paper address subitem 4b-ii?

Copy and paste relevant sections from the manuscript (include quotes in quotation marks "like this" to indicate direct quotes from your manuscript), or elaborate on this item by providing additional information not in the ms, or briefly explain why the item is not applicable/relevant for your study

No institutional affiliations were involved/displayed.

5) The interventions for each group with sufficient details to allow replication, including how and when they were actually administered

5-i) Mention names, credential, affiliations of the developers, sponsors, and owners

Mention names, credential, affiliations of the developers, sponsors, and owners [6] (if authors/evaluators are owners or developer of the software, this needs to be declared in a “Conflict of interest” section or mentioned elsewhere in the manuscript).

	1	2	3	4	5	
subitem not at all important	○	◉	○	○	○	essential

Clear selection

Does your paper address subitem 5-i?

Copy and paste relevant sections from the manuscript (include quotes in quotation marks "like this" to indicate direct quotes from your manuscript), or elaborate on this item by providing additional information not in the ms, or briefly explain why the item is not applicable/relevant for your study

The intervention/program is described in more detail in the published protocol, though names and affiliations of the developers are not noted as this information is not as applicable to the Happify for Teens product given how it has been designed. The study authors are employed by the sponsor (Twill) and are named with credentials, and conflicts of interest are described in the relevant section of the manuscript.

5-ii) Describe the history/development process

Describe the history/development process of the application and previous formative evaluations (e.g., focus groups, usability testing), as these will have an impact on adoption/use rates and help with interpreting results.

	1	2	3	4	5	
subitem not at all important	○	○	◉	○	○	essential

Clear selection

Does your paper address subitem 5-ii?

Copy and paste relevant sections from the manuscript (include quotes in quotation marks "like this" to indicate direct quotes from your manuscript), or elaborate on this item by providing additional information not in the ms, or briefly explain why the item is not applicable/relevant for your study

Details of the intervention are described in the published protocol and this is referenced in this results manuscript (from the published protocol: "Participants assigned to the active intervention will receive instructions to download Happify Health and then be directed into the Teens platform after creating an account. The Happify for Teens platform was developed using the same model as the original Happify Health platform; intervention activities are organized into 6 different skills: savor (mindfulness skills), thank (gratitude), aspire (optimism, goal-setting, and finding meaning or purpose), give (kindness, forgiveness, and prosocial behavior), empathize (self-compassion and perspective-taking), and revive (physical health). However, for the adolescent platform, these activities were modified and then reviewed by a panel of adolescents to ensure that the language and content were relevant to that population").

5-iii) Revisions and updating

Revisions and updating. Clearly mention the date and/or version number of the application/intervention (and comparator, if applicable) evaluated, or describe whether the intervention underwent major changes during the evaluation process, or whether the development and/or content was “frozen” during the trial. Describe dynamic components such as news feeds or changing content which may have an impact on the replicability of the intervention (for unexpected events see item 3b).

	1	2	3	4	5	
subitem not at all important	◉	○	○	○	○	essential

Clear selection

Does your paper address subitem 5-iii?

Copy and paste relevant sections from the manuscript (include quotes in quotation marks "like this" to indicate direct quotes from your manuscript), or elaborate on this item by providing additional information not in the ms, or briefly explain why the item is not applicable/relevant for your study

The intervention was static during the trial and has not been revised since the trial.

5-iv) Quality assurance methods

Provide information on quality assurance methods to ensure accuracy and quality of information provided [1], if applicable.

	1	2	3	4	5	
subitem not at all important	(●)	( )	( )	( )	( )	essential

Clear selection

Does your paper address subitem 5-iv?

Copy and paste relevant sections from the manuscript (include quotes in quotation marks "like this" to indicate direct quotes from your manuscript), or elaborate on this item by providing additional information not in the ms, or briefly explain why the item is not applicable/relevant for your study

This is not addressed in the manuscript.

5-v) Ensure replicability by publishing the source code, and/or providing screenshots/screen-capture video, and/or providing flowcharts of the algorithms used

Ensure replicability by publishing the source code, and/or providing screenshots/screen-capture video, and/or providing flowcharts of the algorithms used. Replicability (i.e., other researchers should in principle be able to replicate the study) is a hallmark of scientific reporting.

	1	2	3	4	5	
subitem not at all important	(•)	( )	( )	( )	( )	essential

Clear selection

Does your paper address subitem 5-v?

Copy and paste relevant sections from the manuscript (include quotes in quotation marks "like this" to indicate direct quotes from your manuscript), or elaborate on this item by providing additional information not in the ms, or briefly explain why the item is not applicable/relevant for your study

Screenshots of the intervention are provided in the published protocol; however, the intervention and its components are proprietary and cannot be shared publicly. Detailed information for the purposes of replicating the study (with the exception of replicating the intervention) are provided in the published protocol.

5-vi) Digital preservation

Digital preservation: Provide the URL of the application, but as the intervention is likely to change or disappear over the course of the years; also make sure the intervention is archived (Internet Archive, webcitation.org, and/or publishing the source code or screenshots/videos alongside the article). As pages behind login screens cannot be archived, consider creating demo pages which are accessible without login.

	1	2	3	4	5	
subitem not at all important	(•)	( )	( )	( )	( )	essential

Clear selection

Does your paper address subitem 5-vi?

Copy and paste relevant sections from the manuscript (include quotes in quotation marks "like this" to indicate direct quotes from your manuscript), or elaborate on this item by providing additional information not in the ms, or briefly explain why the item is not applicable/relevant for your study

The application is not publicly available for adolescents and cannot be accessed directly via URL.

5-vii) Access

Access: Describe how participants accessed the application, in what setting/context, if they had to pay (or were paid) or not, whether they had to be a member of specific group. If known, describe how participants obtained "access to the platform and Internet" [1]. To ensure access for editors/reviewers/readers, consider to provide a "backdoor" login account or demo mode for reviewers/readers to explore the application (also important for archiving purposes, see vi).

	1	2	3	4	5	
subitem not at all important	○	○	●	○	○	essential

Clear selection

Does your paper address subitem 5-vii? *

Copy and paste relevant sections from the manuscript (include quotes in quotation marks "like this" to indicate direct quotes from your manuscript), or elaborate on this item by providing additional information not in the ms, or briefly explain why the item is not applicable/relevant for your study

"Those assigned to the intervention condition received instructions to download the Happify mobile application and were directed into the Happify for Teens platform after creating an account (a detailed description of the intervention was previously published [51])."

5-viii) Mode of delivery, features/functionalities/components of the intervention and comparator, and the theoretical framework

Describe mode of delivery, features/functionalities/components of the intervention and comparator, and the theoretical framework [6] used to design them (instructional strategy [1], behaviour change techniques, persuasive features, etc., see e.g., [7, 8] for terminology). This includes an in-depth description of the content (including where it is coming from and who developed it) [1]," whether [and how] it is tailored to individual circumstances and allows users to track their progress and receive feedback" [6]. This also includes a description of communication delivery channels and – if computer-mediated communication is a component – whether communication was synchronous or asynchronous [6]. It also includes information on presentation strategies [1], including page design principles, average amount of text on pages, presence of hyperlinks to other resources, etc. [1].

	1	2	3	4	5	
subitem not at all important	○	◉	○	○	○	essential

Clear selection

Does your paper address subitem 5-viii? *

Copy and paste relevant sections from the manuscript (include quotes in quotation marks "like this" to indicate direct quotes from your manuscript), or elaborate on this item by providing additional information not in the ms, or briefly explain why the item is not applicable/relevant for your study

The intervention is described in detail in the published protocol paper, which is referenced in the results paper. From the published protocol: "The Happify for Teens platform was developed using the same model as the original Happify Health platform; intervention activities are organized into 6 different skills: savor (mindfulness skills), thank (gratitude), aspire (optimism, goal-setting, and finding meaning or purpose), give (kindness, forgiveness, and prosocial behavior), empathize (self-compassion and perspective-taking), and revive (physical health). However, for the adolescent platform, these activities were modified and then reviewed by a panel of adolescents to ensure that the language and content were relevant to that population. Activities from different categories are then organized into tracks focused on addressing a specific area of concern, such as increasing confidence or reducing negative thinking (see Figure 2 for a sample track description). Users can select a track that interests them, and within each track, they can choose what activities they prefer to complete, thereby offering users a more personalized experience in terms of selecting tracks as well as activities, which has been shown to improve efficacy [101,102]. Although participants will have access to all Happify for Teen tracks, we will feature a track called Stop the Worry Cycle. Each track consists of 4 different parts, and users must complete activities within each part to earn a silver or gold medal before they can move on to the next part. Users can also complete activities on demand through instant play and can switch tracks at any time."

5-ix) Describe use parameters

Describe use parameters (e.g., intended “doses” and optimal timing for use). Clarify what instructions or recommendations were given to the user, e.g., regarding timing, frequency, heaviness of use, if any, or was the intervention used ad libitum.

	1	2	3	4	5	
subitem not at all important	○	○	○	◉	○	essential

Clear selection

Does your paper address subitem 5-ix?

Copy and paste relevant sections from the manuscript (include quotes in quotation marks "like this" to indicate direct quotes from your manuscript), or elaborate on this item by providing additional information not in the ms, or briefly explain why the item is not applicable/relevant for your study

The intervention does not indicate intended doses or optimal timing for use; however, instructions to participants within the context of the trial are described in the manuscript: "Participants received no instructions on how much to engage with the intervention but received push notifications and weekly engagement emails as part of the intervention. We also contacted participants who completed no activities over 7 days via email or text message (based on participant preference). Participants had access to the intervention for the full study period but stopped receiving push notifications or emails regarding usage after 8 weeks."

5-x) Clarify the level of human involvement

Clarify the level of human involvement (care providers or health professionals, also technical assistance) in the e-intervention or as co-intervention (detail number and expertise of professionals involved, if any, as well as “type of assistance offered, the timing and frequency of the support, how it is initiated, and the medium by which the assistance is delivered”. It may be necessary to distinguish between the level of human involvement required for the trial, and the level of human involvement required for a routine application outside of a RCT setting (discuss under item 21 – generalizability).

	1	2	3	4	5	
subitem not at all important	(selected)	○	○	○	○	essential

Clear selection

Does your paper address subitem 5-x?

Copy and paste relevant sections from the manuscript (include quotes in quotation marks "like this" to indicate direct quotes from your manuscript), or elaborate on this item by providing additional information not in the ms, or briefly explain why the item is not applicable/relevant for your study

The intervention is described as self-guided throughout the manuscript and included no human involvement.

5-xi) Report any prompts/reminders used

Report any prompts/reminders used: Clarify if there were prompts (letters, emails, phone calls, SMS) to use the application, what triggered them, frequency etc. It may be necessary to distinguish between the level of prompts/reminders required for the trial, and the level of prompts/reminders for a routine application outside of a RCT setting (discuss under item 21 – generalizability).

	1	2	3	4	5	
subitem not at all important	○	○	○	◉	○	essential

Clear selection

Does your paper address subitem 5-xi? *

Copy and paste relevant sections from the manuscript (include quotes in quotation marks "like this" to indicate direct quotes from your manuscript), or elaborate on this item by providing additional information not in the ms, or briefly explain why the item is not applicable/relevant for your study

Prompts that were part of the intervention itself as well as dedicated prompts from the research team are described in the manuscript: "Participants received no instructions on how much to engage with the intervention but received push notifications and weekly engagement emails as part of the intervention. We also contacted participants who completed no activities over 7 days via email or text message (based on participant preference). Participants had access to the intervention for the full study period but stopped receiving push notifications or emails regarding usage after 8 weeks."

5-xii) Describe any co-interventions (incl. training/support)

Describe any co-interventions (incl. training/support): Clearly state any interventions that are provided in addition to the targeted eHealth intervention, as ehealth intervention may not be designed as stand-alone intervention. This includes training sessions and support [1]. It may be necessary to distinguish between the level of training required for the trial, and the level of training for a routine application outside of a RCT setting (discuss under item 21 – generalizability.

	1	2	3	4	5	
subitem not at all important	◉	○	○	○	○	essential

Clear selection

Does your paper address subitem 5-xii? *

Copy and paste relevant sections from the manuscript (include quotes in quotation marks "like this" to indicate direct quotes from your manuscript), or elaborate on this item by providing additional information not in the ms, or briefly explain why the item is not applicable/relevant for your study

There were no co-interventions involved, or training/support.

6a) Completely defined pre-specified primary and secondary outcome measures, including how and when they were assessed

Does your paper address CONSORT subitem 6a? *

Copy and paste relevant sections from the manuscript (include quotes in quotation marks "like this" to indicate direct quotes from your manuscript), or elaborate on this item by providing additional information not in the ms, or briefly explain why the item is not applicable/relevant for your study

"Primary Outcome: Perceived Stress. The PSS [52] consists of 10 items asking participants how often they felt each feeling/thought in the previous month (e.g., "how often have you felt nervous and "stressed"?"). Items are rated on a scale from 0 (never) to 4 (very often), and ratings are summed so higher scores indicate greater perceived stress. Across the four time points, internal reliabilities ranged from acceptable to good (αs:.77-.88).
Secondary Outcome: Brooding. The Brooding Subscale of the RRS [53] asks respondents to indicate how often they engage in five behaviors (e.g., "Think 'Why can't I handle things better'?") on a scale from 1 (almost never) to 4 (almost always). Participants in the current study were asked to reflect on the previous month. Ratings are summed so higher scores indicate more brooding. Internal reliability values were fair to acceptable (αs:.61-.78)." and timing is described in the procedure: "Every four weeks, participants were prompted to complete outcome measures via Qualtrics. Participants were contacted via email or text message (based on participant preference) if an assessment was not completed within 7 days of its scheduled date."

6a-i) Online questionnaires: describe if they were validated for online use and apply CHERRIES items to describe how the questionnaires were designed/deployed

If outcomes were obtained through online questionnaires, describe if they were validated for online use and apply CHERRIES items to describe how the questionnaires were designed/deployed [9].

	1	2	3	4	5	
subitem not at all important	(•)	( )	( )	( )	( )	essential

Clear selection

Does your paper address subitem 6a-i?

Copy and paste relevant sections from manuscript text

Questionnaires were not specifically validated for online use, but are standard, widely used measures that have been administered online via Qualtrics for other research. Measures were not modified in any way and merely imported to the Qualtrics survey tool.

6a-ii) Describe whether and how "use" (including intensity of use/dosage) was defined/measured/monitored

Describe whether and how "use" (including intensity of use/dosage) was defined/measured/monitored (logins, logfile analysis, etc.). Use/adoption metrics are important process outcomes that should be reported in any ehealth trial.

	1	2	3	4	5	
subitem not at all important	◉	○	○	○	○	essential

Clear selection

Does your paper address subitem 6a-ii?

Copy and paste relevant sections from manuscript text

Given the comparison to a waitlist control, usage was not included as a variable of interest and thus intensity of use/dosage was not defined. Activity completion within the intervention for participants assigned to the intervention condition was measured by the application in order to determine whether the participant had been exposed to the intervention or not, and for tracking purposes to send reminders to participants to engage with the intervention, as described in the manuscript: "We also contacted participants who completed no activities over 7 days via email or text message (based on participant preference)."

6a-iii) Describe whether, how, and when qualitative feedback from participants was obtained

Describe whether, how, and when qualitative feedback from participants was obtained (e.g., through emails, feedback forms, interviews, focus groups).

	1	2	3	4	5	
subitem not at all important	(●)	( )	( )	( )	( )	essential

Clear selection

Does your paper address subitem 6a-iii?

Copy and paste relevant sections from manuscript text

Although a qualitative follow-up was conducted with a subset of trial participants, qualitative feedback was not part of the main trial and is not reported in this manuscript.

6b) Any changes to trial outcomes after the trial commenced, with reasons

Does your paper address CONSORT subitem 6b? *

Copy and paste relevant sections from the manuscript (include quotes in quotation marks "like this" to indicate direct quotes from your manuscript), or elaborate on this item by providing additional information not in the ms, or briefly explain why the item is not applicable/relevant for your study

No changes to the trial outcomes were made after the trial commenced, or after the protocol was published.

7a) How sample size was determined

NPT: When applicable, details of whether and how the clustering by care provides or centers was addressed

7a-i) Describe whether and how expected attrition was taken into account when calculating the sample size

Describe whether and how expected attrition was taken into account when calculating the sample size.

	1	2	3	4	5	
subitem not at all important	○	○	○	◉	○	essential

Clear selection

Does your paper address subitem 7a-i?

Copy and paste relevant sections from manuscript title (include quotes in quotation marks "like this" to indicate direct quotes from your manuscript), or elaborate on this item by providing additional information not in the ms, or briefly explain why the item is not applicable/relevant for your study

Sample size determination is described in more detail in the published protocol; however, deviations from the original target sample size - which accounted for predicted attrition - is described in this manuscript: "Note that this sample is smaller than the target sample size reported in the published protocol [51]. As described in that protocol, however, power analyses indicated that 200 participants was sufficient for 80% power to detect a small effect for our primary outcome. Due to the high levels of dropout observed with digital interventions [58], including with adolescents, we assumed a 75% attrition rate and set a target sample size of 800 participants. Because attrition rates were much lower than expected (assessment completion rates ranged from 78.95% to 87.13%), we decided to stop enrollment once 100 participants per condition completed the 12-week assessment to avoid oversampling, resulting in the smaller sample."

7b) When applicable, explanation of any interim analyses and stopping guidelines

Does your paper address CONSORT subitem 7b? *

Copy and paste relevant sections from the manuscript (include quotes in quotation marks "like this" to indicate direct quotes from your manuscript), or elaborate on this item by providing additional information not in the ms, or briefly explain why the item is not applicable/relevant for your study

Not applicable; stopping guidelines are described in reference to sample size - specifically that due to lower attrition rates than expected, recruitment was stopped once we achieved 100 participants per condition who completed the study (the sample size identified in power analyses).

8a) Method used to generate the random allocation sequence

NPT: When applicable, how care providers were allocated to each trial group

Does your paper address CONSORT subitem 8a? *

Copy and paste relevant sections from the manuscript (include quotes in quotation marks "like this" to indicate direct quotes from your manuscript), or elaborate on this item by providing additional information not in the ms, or briefly explain why the item is not applicable/relevant for your study

"Once enrolled, participants were randomized to condition; randomization was completed automatically by Qualtrics using block randomization upon completing the baseline assessment and meeting final eligibility criteria."

8b) Type of randomisation; details of any restriction (such as blocking and block size)

Does your paper address CONSORT subitem 8b? *

Copy and paste relevant sections from the manuscript (include quotes in quotation marks "like this" to indicate direct quotes from your manuscript), or elaborate on this item by providing additional information not in the ms, or briefly explain why the item is not applicable/relevant for your study

"Once enrolled, participants were randomized to condition; randomization was completed automatically by Qualtrics using block randomization upon completing the baseline assessment and meeting final eligibility criteria."

9) Mechanism used to implement the random allocation sequence (such as sequentially numbered containers), describing any steps taken to conceal the sequence until interventions were assigned

Does your paper address CONSORT subitem 9? *

Copy and paste relevant sections from the manuscript (include quotes in quotation marks "like this" to indicate direct quotes from your manuscript), or elaborate on this item by providing additional information not in the ms, or briefly explain why the item is not applicable/relevant for your study

As described in the manuscript, randomization was performed automatically by the Qualtrics program once a participant completed the baseline assessment and met eligibility criteria - information on the random allocation sequence is not available, but as described in the manuscript, block randomization was used to ensure equivalent groups.

10) Who generated the random allocation sequence, who enrolled participants, and who assigned participants to interventions

Does your paper address CONSORT subitem 10? *

Copy and paste relevant sections from the manuscript (include quotes in quotation marks "like this" to indicate direct quotes from your manuscript), or elaborate on this item by providing additional information not in the ms, or briefly explain why the item is not applicable/relevant for your study

As described in the manuscript, randomization was performed automatically by Qualtrics.

11a) If done, who was blinded after assignment to interventions (for example, participants, care providers, those assessing outcomes) and how

NPT: Whether or not administering co-interventions were blinded to group assignment

11a-i) Specify who was blinded, and who wasn't

Specify who was blinded, and who wasn't. Usually, in web-based trials it is not possible to blind the participants [1, 3] (this should be clearly acknowledged), but it may be possible to blind outcome assessors, those doing data analysis or those administering co-interventions (if any).

	1	2	3	4	5	
subitem not at all important	(●)	( )	( )	( )	( )	essential

Clear selection

Does your paper address subitem 11a-i? *

Copy and paste relevant sections from the manuscript (include quotes in quotation marks "like this" to indicate direct quotes from your manuscript), or elaborate on this item by providing additional information not in the ms, or briefly explain why the item is not applicable/relevant for your study

Given the waitlist control group, this trial was not blinded as the research team had to contact participants in the intervention condition in regards to usage; invitations to complete assessments was completed automatically via Qualtrics using the participants' email and was collected without information about condition (with the exception of the baseline assessments). Assessments were merged for data analysis using a participant ID, which was entered for every assessment period, at which time data was identifiable by condition.

11a-ii) Discuss e.g., whether participants knew which intervention was the “intervention of interest” and which one was the “comparator”

Informed consent procedures (4a-ii) can create biases and certain expectations - discuss e.g., whether participants knew which intervention was the “intervention of interest” and which one was the “comparator”.

	1	2	3	4	5	
subitem not at all important	(selected)					essential

Clear selection

Does your paper address subitem 11a-ii?

Copy and paste relevant sections from the manuscript (include quotes in quotation marks "like this" to indicate direct quotes from your manuscript), or elaborate on this item by providing additional information not in the ms, or briefly explain why the item is not applicable/relevant for your study

The comparator was a waitlist control and, consequently, participants clearly understood what was the intervention of interest.

11b) If relevant, description of the similarity of interventions
(this item is usually not relevant for ehealth trials as it refers to similarity of a placebo or sham intervention to a active medication/intervention)

Does your paper address CONSORT subitem 11b? *

Copy and paste relevant sections from the manuscript (include quotes in quotation marks "like this" to indicate direct quotes from your manuscript), or elaborate on this item by providing additional information not in the ms, or briefly explain why the item is not applicable/relevant for your study

Not applicable; the comparator was a waitlist control.

12a) Statistical methods used to compare groups for primary and secondary outcomes
NPT: When applicable, details of whether and how the clustering by care providers or centers was addressed

Does your paper address CONSORT subitem 12a? *

Copy and paste relevant sections from the manuscript (include quotes in quotation marks "like this" to indicate direct quotes from your manuscript), or elaborate on this item by providing additional information not in the ms, or briefly explain why the item is not applicable/relevant for your study

"To assess whether changes in outcomes differed across groups, we created repeated-measures multilevel models (MLMs) for each outcome. MLMs were computed in R using the lme4 package [57] and restricted maximum likelihood estimation. All models included a random slope for time (Δχ2s≥10.35, ps≤.006) and cross-level interactions for condition (waitlist; intervention) x time (baseline; 4-weeks; 8-weeks; 12-weeks). We examined regression assumptions (e.g., residual normality, influential observations), and final models were run with the small number of influential outliers removed (2-4, depending on outcome). In addition, because careless responding is problematic with online surveys and may artificially increase relationships between variables, we used two a priori mechanisms for identifying low quality data: (1) failing three or more attention checks in any given assessment, and (2) completing an assessment at a rate faster than one second per item [59]. Specific assessments that met either of these criteria were dropped rather than dropping the participant altogether, and analyses were re-run without these assessments as a sensitivity analysis."

12a-i) Imputation techniques to deal with attrition / missing values

Imputation techniques to deal with attrition / missing values: Not all participants will use the intervention/comparator as intended and attrition is typically high in ehealth trials. Specify how participants who did not use the application or dropped out from the trial were treated in the statistical analysis (a complete case analysis is strongly discouraged, and simple imputation techniques such as LOCF may also be problematic [4]).

	1	2	3	4	5	
subitem not at all important	○	○	○	●	○	essential

Clear selection

Does your paper address subitem 12a-i? *

Copy and paste relevant sections from the manuscript (include quotes in quotation marks "like this" to indicate direct quotes from your manuscript), or elaborate on this item by providing additional information not in the ms, or briefly explain why the item is not applicable/relevant for your study

Data was not imputed for data analyses, as described in the manuscript: "Of the 331 participants who completed the study, 28 were excluded from data analyses (see Figure 1). Of note, 22 participants in the intervention condition failed to complete any activities throughout the study period (and thus were not exposed to the intervention). Five participants were excluded for providing odd or missing birth years on the baseline assessment, and one additional participant was excluded for having duplicate responses to assessments. This resulted in a total of 303 participants included in our analyses (Happify for Teens: n = 132; Waitlist: n = 171)." Participants who did not provide some assessments were still included in the multilevel models, which can account for missing data without imputation.

12b) Methods for additional analyses, such as subgroup analyses and adjusted analyses

Does your paper address CONSORT subitem 12b? *

Copy and paste relevant sections from the manuscript (include quotes in quotation marks "like this" to indicate direct quotes from your manuscript), or elaborate on this item by providing additional information not in the ms, or briefly explain why the item is not applicable/relevant for your study

Subgroup analyses were not performed; however, sensitivity analyses were performed removing participants who had failed a priori criteria for attention checks, as described in the published protocol and the manuscript: "In addition, because careless responding is problematic with online surveys and may artificially increase relationships between variables, we used two a priori mechanisms for identifying low quality data: (1) failing three or more attention checks in any given assessment, and (2) completing an assessment at a rate faster than one second per item [59]. Specific assessments that met either of these criteria were dropped rather than dropping the participant altogether, and analyses were re-run without these assessments as a sensitivity analysis." The outcomes relevant to this sensitivity analyses are also described: "Only four participants were flagged for attention checks (one on three of four assessments, one on two assessments, and two on just one assessment). No participants were flagged for completion rate. Primary and secondary outcomes did not change meaningfully in these analyses when these assessments were excluded (but see Footnote 2)."

X26) REB/IRB Approval and Ethical Considerations [recommended as subheading under "Methods"] (not a CONSORT item)

X26-i) Comment on ethics committee approval

	1	2	3	4	5	
subitem not at all important	○	○	○	○	◉	essential

Clear selection

Does your paper address subitem X26-i?

Copy and paste relevant sections from the manuscript (include quotes in quotation marks "like this" to indicate direct quotes from your manuscript), or elaborate on this item by providing additional information not in the ms, or briefly explain why the item is not applicable/relevant for your study

"Study procedures were reviewed and approved by IntegReview, an independent IRB, which was acquired by Advarra after the study was launched."

x26-ii) Outline informed consent procedures

Outline informed consent procedures e.g., if consent was obtained offline or online (how? Checkbox, etc.?), and what information was provided (see 4a-ii). See [6] for some items to be included in informed consent documents.

	1	2	3	4	5	
subitem not at all important	○	○	○	●	○	essential

Clear selection

Does your paper address subitem X26-ii?

Copy and paste relevant sections from the manuscript (include quotes in quotation marks "like this" to indicate direct quotes from your manuscript), or elaborate on this item by providing additional information not in the ms, or briefly explain why the item is not applicable/relevant for your study

Due to the nature of using adolescents, the consent and assent process are referenced briefly in the manuscript: "and to provide electronic consent for each eligible child" and "Eligible participants were directed to complete the baseline assessment and provide electronic assent to participate in the study."

X26-iii) Safety and security procedures

Safety and security procedures, incl. privacy considerations, and any steps taken to reduce the likelihood or detection of harm (e.g., education and training, availability of a hotline)

	1	2	3	4	5	
subitem not at all important	○	◉	○	○	○	essential

Clear selection

Does your paper address subitem X26-iii?

Copy and paste relevant sections from the manuscript (include quotes in quotation marks "like this" to indicate direct quotes from your manuscript), or elaborate on this item by providing additional information not in the ms, or briefly explain why the item is not applicable/relevant for your study

Safety and security procedures were covered by the standard terms and conditions (including the privacy policy) outlined for Happify for Teens. Specific privacy considerations relevant to the trial were described in the consent and assent forms, but are not described in the manuscript.

RESULTS

13a) For each group, the numbers of participants who were randomly assigned, received intended treatment, and were analysed for the primary outcome

NPT: The number of care providers or centers performing the intervention in each group and the number of patients treated by each care provider in each center

Does your paper address CONSORT subitem 13a? *

Copy and paste relevant sections from the manuscript (include quotes in quotation marks "like this" to indicate direct quotes from your manuscript), or elaborate on this item by providing additional information not in the ms, or briefly explain why the item is not applicable/relevant for your study

"Of the 331 participants who completed the study, 28 were excluded from data analyses (see Figure 1). Of note, 22 participants in the intervention condition failed to complete any activities throughout the study period (and thus were not exposed to the intervention). Five participants were excluded for providing odd or missing birth years on the baseline assessment, and one additional participant was excluded for having duplicate responses to assessments. This resulted in a total of 303 participants included in our analyses (Happify for Teens: n = 132; Waitlist: n = 171)." This information is also presented in a full CONSORT diagram.

13b) For each group, losses and exclusions after randomisation, together with reasons

Does your paper address CONSORT subitem 13b? (NOTE: Preferably, this is shown in a CONSORT flow diagram) *

Copy and paste relevant sections from the manuscript (include quotes in quotation marks "like this" to indicate direct quotes from your manuscript), or elaborate on this item by providing additional information not in the ms, or briefly explain why the item is not applicable/relevant for your study

"Of the 331 participants who completed the study, 28 were excluded from data analyses (see Figure 1). Of note, 22 participants in the intervention condition failed to complete any activities throughout the study period (and thus were not exposed to the intervention). Five participants were excluded for providing odd or missing birth years on the baseline assessment, and one additional participant was excluded for having duplicate responses to assessments. This resulted in a total of 303 participants included in our analyses (Happify for Teens: n = 132; Waitlist: n = 171)." This information is also presented in a full CONSORT diagram.

13b-i) Attrition diagram

Strongly recommended: An attrition diagram (e.g., proportion of participants still logging in or using the intervention/comparator in each group plotted over time, similar to a survival curve) or other figures or tables demonstrating usage/dose/engagement.

	1	2	3	4	5	
subitem not at all important	○	○	○	○	◉	essential

Clear selection

Does your paper address subitem 13b-i?

Copy and paste relevant sections from the manuscript or cite the figure number if applicable (include quotes in quotation marks "like this" to indicate direct quotes from your manuscript), or elaborate on this item by providing additional information not in the ms, or briefly explain why the item is not applicable/relevant for your study

A full CONSORT diagram is presented in the manuscript

14a) Dates defining the periods of recruitment and follow-up

Does your paper address CONSORT subitem 14a? *

Copy and paste relevant sections from the manuscript (include quotes in quotation marks "like this" to indicate direct quotes from your manuscript), or elaborate on this item by providing additional information not in the ms, or briefly explain why the item is not applicable/relevant for your study

Periods of recruitment and enrollment are described in the manuscript: "Study enrollment began on June 3, 2021 and continued until December 18, 2021."

14a-i) Indicate if critical “secular events” fell into the study period

Indicate if critical “secular events” fell into the study period, e.g., significant changes in Internet resources available or “changes in computer hardware or Internet delivery resources”

	1	2	3	4	5	
subitem not at all important	(●)	○	○	○	○	essential

Clear selection

Does your paper address subitem 14a-i?

Copy and paste relevant sections from the manuscript (include quotes in quotation marks "like this" to indicate direct quotes from your manuscript), or elaborate on this item by providing additional information not in the ms, or briefly explain why the item is not applicable/relevant for your study

To our knowledge, no critical "secular events" fell into the study period.

14b) Why the trial ended or was stopped (early)

Does your paper address CONSORT subitem 14b? *

Copy and paste relevant sections from the manuscript (include quotes in quotation marks "like this" to indicate direct quotes from your manuscript), or elaborate on this item by providing additional information not in the ms, or briefly explain why the item is not applicable/relevant for your study

The trial was not ended early; however, a smaller sample was recruited than originally intended due to lower attrition than expected, as described in the manuscript: "Note that this sample is smaller than the target sample size reported in the published protocol [51]. As described in that protocol, however, power analyses indicated that 200 participants was sufficient for 80% power to detect a small effect for our primary outcome. Due to the high levels of dropout observed with digital interventions [58], including with adolescents, we assumed a 75% attrition rate and set a target sample size of 800 participants. Because attrition rates were much lower than expected (assessment completion rates ranged from 78.95% to 87.13%), we decided to stop enrollment once 100 participants per condition completed the 12-week assessment to avoid oversampling, resulting in the smaller sample."

15) A table showing baseline demographic and clinical characteristics for each group

NPT: When applicable, a description of care providers (case volume, qualification, expertise, etc.) and centers (volume) in each group

Does your paper address CONSORT subitem 15? *

Copy and paste relevant sections from the manuscript (include quotes in quotation marks "like this" to indicate direct quotes from your manuscript), or elaborate on this item by providing additional information not in the ms, or briefly explain why the item is not applicable/relevant for your study

Demographic characteristics for the analytical sample are displayed in Table 1; descriptives for all outcomes at each assessment period are also displayed in Table 2.

15-i) Report demographics associated with digital divide issues

In ehealth trials it is particularly important to report demographics associated with digital divide issues, such as age, education, gender, social-economic status, computer/Internet/ehealth literacy of the participants, if known.

	1	2	3	4	5	
subitem not at all important	○	○	○	○	◉	essential

Clear selection

Does your paper address subitem 15-i? *

Copy and paste relevant sections from the manuscript (include quotes in quotation marks "like this" to indicate direct quotes from your manuscript), or elaborate on this item by providing additional information not in the ms, or briefly explain why the item is not applicable/relevant for your study

Gender, Age, and Race/Ethnicity are reported in Table 1; as the sample was adolescents, education information was not collected but a question regarding current schooling situation was included in the baseline assessment and descriptive statistics for this item are also presented in Table 1. Socioeconomic status and computer/Internet/ehealth literacy information was not collected.

16) For each group, number of participants (denominator) included in each analysis and whether the analysis was by original assigned groups

16-i) Report multiple “denominators” and provide definitions

Report multiple “denominators” and provide definitions: Report N’s (and effect sizes) “across a range of study participation [and use] thresholds” [1], e.g., N exposed, N consented, N used more than x times, N used more than y weeks, N participants “used” the intervention/comparator at specific pre-defined time points of interest (in absolute and relative numbers per group). Always clearly define “use” of the intervention.

	1	2	3	4	5	
subitem not at all important	○	○	◉	○	○	essential

Clear selection

Does your paper address subitem 16-i? *

Copy and paste relevant sections from the manuscript (include quotes in quotation marks "like this" to indicate direct quotes from your manuscript), or elaborate on this item by providing additional information not in the ms, or briefly explain why the item is not applicable/relevant for your study

Multiple denominators were not used for analytical purposes; however, a full participant flow for the study - including the original group sizes, participants who assented, engaged with the intervention etc - are displayed in the CONSORT diagram in Figure 1, and reasons for data exclusion are also described in the manuscript.

16-ii) Primary analysis should be intent-to-treat

Primary analysis should be intent-to-treat, secondary analyses could include comparing only “users”, with the appropriate caveats that this is no longer a randomized sample (see 18-i).

	1	2	3	4	5	
subitem not at all important	○	○	○	○	○	essential

Does your paper address subitem 16-ii?

Copy and paste relevant sections from the manuscript (include quotes in quotation marks "like this" to indicate direct quotes from your manuscript), or elaborate on this item by providing additional information not in the ms, or briefly explain why the item is not applicable/relevant for your study

22 participants from the intervention condition were dropped from analyses for not completing any activities whatsoever during the 12-week study period. This decision was made a priori (before conducting any data analyses) because these participants were not exposed to the intervention and thus were equivalent to the waitlist control (who were not exposed to any intervention), unlike a study with an active control where participants in both the intervention and placebo conditions may have no engagement and not be exposed to their respective "interventions".

17a) For each primary and secondary outcome, results for each group, and the estimated effect size and its precision (such as 95% confidence interval)

Does your paper address CONSORT subitem 17a? *

Copy and paste relevant sections from the manuscript (include quotes in quotation marks "like this" to indicate direct quotes from your manuscript), or elaborate on this item by providing additional information not in the ms, or briefly explain why the item is not applicable/relevant for your study

Primary Outcome: "We found a significant main effect of time ($p < .001$) but no significant effect of condition ($p = .968$). These were qualified by the predicted condition x time interaction ($p < .001$). To break down this interaction, we calculated simple slopes for each condition, which revealed a significant reduction in perceived stress among participants in the intervention condition (B = -1.50, 95% CI: -1.82 - -1.19; $p < .001$) but not among those in the waitlist control (B = -.09, 95% CI: -0.44 – 0.26; $p = .607$) (See Figure 2)."

Secondary Outcome: "We also found a significant effect of time ($p < .001$) but not of condition ($p = .154$) for brooding. These effects were qualified by a significant condition x time interaction ($p < .001$). There were significant reductions in brooding among participants in the waitlist control (B = -0.30, 95% CI: -0.47 - -0.12; $p = .001$) as well as those in the Happify for Teens condition (B = -0.84, 95% CI: -1.00 - -0.68; $p < .001$); however, confidence intervals for the two groups did not overlap, suggesting that improvements were relatively greater among those in the intervention condition (see Figure 3). Again, with the exception of a significant effect of gender such that participants identifying as male reported significantly lower levels of brooding ($p < .001$), no other effects were significant (ps ≥ .353)."

17a-i) Presentation of process outcomes such as metrics of use and intensity of use

In addition to primary/secondary (clinical) outcomes, the presentation of process outcomes such as metrics of use and intensity of use (dose, exposure) and their operational definitions is critical. This does not only refer to metrics of attrition (13-b) (often a binary variable), but also to more continuous exposure metrics such as "average session length". These must be accompanied by a technical description how a metric like a "session" is defined (e.g., timeout after idle time) [1] (report under item 6a).

	1	2	3	4	5	
subitem not at all important	◉	○	○	○	○	essential

Clear selection

Does your paper address subitem 17a-i?

Copy and paste relevant sections from the manuscript (include quotes in quotation marks "like this" to indicate direct quotes from your manuscript), or elaborate on this item by providing additional information not in the ms, or briefly explain why the item is not applicable/relevant for your study

Although originally intended, the impact of usage on outcomes was not examined in this study given that usage was relevant only for the intervention condition and thus the resulting analytical sample would be too small (n = 132) to appropriately explore the impact of usage on outcomes given the known variability in usage metrics.

17b) For binary outcomes, presentation of both absolute and relative effect sizes is recommended

Does your paper address CONSORT subitem 17b? *

Copy and paste relevant sections from the manuscript (include quotes in quotation marks "like this" to indicate direct quotes from your manuscript), or elaborate on this item by providing additional information not in the ms, or briefly explain why the item is not applicable/relevant for your study

No binary outcomes are included in this trial.

18) Results of any other analyses performed, including subgroup analyses and adjusted analyses, distinguishing pre-specified from exploratory

Does your paper address CONSORT subitem 18? *

Copy and paste relevant sections from the manuscript (include quotes in quotation marks "like this" to indicate direct quotes from your manuscript), or elaborate on this item by providing additional information not in the ms, or briefly explain why the item is not applicable/relevant for your study

Exploratory analyses are clearly outlined and described in the manuscript under the Results section.

18-i) Subgroup analysis of comparing only users

A subgroup analysis of comparing only users is not uncommon in ehealth trials, but if done, it must be stressed that this is a self-selected sample and no longer an unbiased sample from a randomized trial (see 16-iii).

	1	2	3	4	5	
subitem not at all important	○	○	○	◉	○	essential

Clear selection

Does your paper address subitem 18-i?

Copy and paste relevant sections from the manuscript (include quotes in quotation marks "like this" to indicate direct quotes from your manuscript), or elaborate on this item by providing additional information not in the ms, or briefly explain why the item is not applicable/relevant for your study

Although we did not conduct a subgroup analysis, our primary analyses excluded users who did not engage with the intervention whatsoever for the full study period. We acknowledge that this is no longer an unbiased sample from a randomized trial in Footnote 2: "The authors acknowledge that by excluding participants who did not engage with the intervention whatsoever during the study period that the sub-sample in the intervention condition is subject to self-selection bias and no longer fully randomized."

19) All important harms or unintended effects in each group

(for specific guidance see CONSORT for harms)

Does your paper address CONSORT subitem 19? *

Copy and paste relevant sections from the manuscript (include quotes in quotation marks "like this" to indicate direct quotes from your manuscript), or elaborate on this item by providing additional information not in the ms, or briefly explain why the item is not applicable/relevant for your study

No harms or unintended effects for either group were observed in this trial.

19-i) Include privacy breaches, technical problems

Include privacy breaches, technical problems. This does not only include physical “harm” to participants, but also incidents such as perceived or real privacy breaches [1], technical problems, and other unexpected/unintended incidents. “Unintended effects” also includes unintended positive effects [2].

	1	2	3	4	5	
subitem not at all important	(selected)	○	○	○	○	essential

Clear selection

Does your paper address subitem 19-i?

Copy and paste relevant sections from the manuscript (include quotes in quotation marks "like this" to indicate direct quotes from your manuscript), or elaborate on this item by providing additional information not in the ms, or briefly explain why the item is not applicable/relevant for your study

No privacy breaches or technical problems occurred during the trial.

19-ii) Include qualitative feedback from participants or observations from staff/researchers

Include qualitative feedback from participants or observations from staff/researchers, if available, on strengths and shortcomings of the application, especially if they point to unintended/unexpected effects or uses. This includes (if available) reasons for why people did or did not use the application as intended by the developers.

	1	2	3	4	5	
subitem not at all important	○	○	(selected)	○	○	essential

Clear selection

Does your paper address subitem 19-ii?

Copy and paste relevant sections from the manuscript (include quotes in quotation marks "like this" to indicate direct quotes from your manuscript), or elaborate on this item by providing additional information not in the ms, or briefly explain why the item is not applicable/relevant for your study

Qualitative feedback was obtained from a subset of trial participants as part of a separate, follow-up study and thus is not reported as part of this trial.

DISCUSSION

22) Interpretation consistent with results, balancing benefits and harms, and considering other relevant evidence

NPT: In addition, take into account the choice of the comparator, lack of or partial blinding, and unequal expertise of care providers or centers in each group

22-i) Restate study questions and summarize the answers suggested by the data, starting with primary outcomes and process outcomes (use)

Restate study questions and summarize the answers suggested by the data, starting with primary outcomes and process outcomes (use).

	1	2	3	4	5	
subitem not at all important	○	○	○	◉	○	essential

Clear selection

Does your paper address subitem 22-i? *

Copy and paste relevant sections from the manuscript (include quotes in quotation marks "like this" to indicate direct quotes from your manuscript), or elaborate on this item by providing additional information not in the ms, or briefly explain why the item is not applicable/relevant for your study

"The purpose of the current study was to test the efficacy of a DMHI designed for adolescents on perceived stress and brooding. Our findings suggest that adolescents who engaged with the intervention had significant reductions in perceived stress, rumination, and loneliness over 12 weeks relative to those in a waitlist control, but not in sleep disturbance or optimism."

22-ii) Highlight unanswered new questions, suggest future research

Highlight unanswered new questions, suggest future research.

	1	2	3	4	5	
subitem not at all important	○	○	○	◉	○	essential

Clear selection

Does your paper address subitem 22-ii?

Copy and paste relevant sections from the manuscript (include quotes in quotation marks "like this" to indicate direct quotes from your manuscript), or elaborate on this item by providing additional information not in the ms, or briefly explain why the item is not applicable/relevant for your study

Limitations and Future Directions are addressed in a dedicated section of the Discussion.

20) Trial limitations, addressing sources of potential bias, imprecision, and, if relevant, multiplicity of analyses

20-i) Typical limitations in ehealth trials

Typical limitations in ehealth trials: Participants in ehealth trials are rarely blinded. Ehealth trials often look at a multiplicity of outcomes, increasing risk for a Type I error. Discuss biases due to non-use of the intervention/usability issues, biases through informed consent procedures, unexpected events.

	1	2	3	4	5	
subitem not at all important	○	○	○	◉	○	essential

Clear selection

Does your paper address subitem 20-i? *

Copy and paste relevant sections from the manuscript (include quotes in quotation marks "like this" to indicate direct quotes from your manuscript), or elaborate on this item by providing additional information not in the ms, or briefly explain why the item is not applicable/relevant for your study

We discuss limitations that are particularly relevant to this trial including digital placebo effects, lack of long-term follow-up, and need for caregiver consent during the recruitment process.

21) Generalisability (external validity, applicability) of the trial findings

NPT: External validity of the trial findings according to the intervention, comparators, patients, and care providers or centers involved in the trial

21-i) Generalizability to other populations

Generalizability to other populations: In particular, discuss generalizability to a general Internet population, outside of a RCT setting, and general patient population, including applicability of the study results for other organizations

	1	2	3	4	5	
subitem not at all important	○	○	○	○	◉	essential

Clear selection

Does your paper address subitem 21-i?

Copy and paste relevant sections from the manuscript (include quotes in quotation marks "like this" to indicate direct quotes from your manuscript), or elaborate on this item by providing additional information not in the ms, or briefly explain why the item is not applicable/relevant for your study

Limitations to generalizability that are particularly relevant to this trial are discussed, including the relative homogeneity of the sample, and how the requirement for caregiver consent may have led to a biased sample that may not generalize to a broader population of adolescents.

21-ii) Discuss if there were elements in the RCT that would be different in a routine application setting

Discuss if there were elements in the RCT that would be different in a routine application setting (e.g., prompts/reminders, more human involvement, training sessions or other co-interventions) and what impact the omission of these elements could have on use, adoption, or outcomes if the intervention is applied outside of a RCT setting.

	1	2	3	4	5	
subitem not at all important	◉	○	○	○	○	essential

Clear selection

Does your paper address subitem 21-ii?

Copy and paste relevant sections from the manuscript (include quotes in quotation marks "like this" to indicate direct quotes from your manuscript), or elaborate on this item by providing additional information not in the ms, or briefly explain why the item is not applicable/relevant for your study

These elements are not discussed as there was little researcher involvement throughout the trial and thus very few differences between a routine application setting and the trial procedures; differences are identified in the Methods.

OTHER INFORMATION

23) Registration number and name of trial registry

Does your paper address CONSORT subitem 23? *

Copy and paste relevant sections from the manuscript (include quotes in quotation marks "like this" to indicate direct quotes from your manuscript), or elaborate on this item by providing additional information not in the ms, or briefly explain why the item is not applicable/relevant for your study

"Clinicaltrials.gov: NCT04567888"

24) Where the full trial protocol can be accessed, if available

Does your paper address CONSORT subitem 24? *

Cite a Multimedia Appendix, other reference, or copy and paste relevant sections from the manuscript (include quotes in quotation marks "like this" to indicate direct quotes from your manuscript), or elaborate on this item by providing additional information not in the ms, or briefly explain why the item is not applicable/relevant for your study

The trial protocol was published in JMIR Protocols and is cited throughout the manuscript.

25) Sources of funding and other support (such as supply of drugs), role of funders

Does your paper address CONSORT subitem 25? *

Copy and paste relevant sections from the manuscript (include quotes in quotation marks "like this" to indicate direct quotes from your manuscript), or elaborate on this item by providing additional information not in the ms, or briefly explain why the item is not applicable/relevant for your study

Under Conflicts of Interest, the study sponsor is identified and authors are described as being employees of the sponsor during the study period.

X27) Conflicts of Interest (not a CONSORT item)

X27-i) State the relation of the study team towards the system being evaluated

In addition to the usual declaration of interests (financial or otherwise), also state the relation of the study team towards the system being evaluated, i.e., state if the authors/evaluators are distinct from or identical with the developers/sponsors of the intervention.

	1	2	3	4	5	
subitem not at all important	○	○	○	○	◉	essential

Clear selection

Does your paper address subitem X27-i?

Copy and paste relevant sections from the manuscript (include quotes in quotation marks "like this" to indicate direct quotes from your manuscript), or elaborate on this item by providing additional information not in the ms, or briefly explain why the item is not applicable/relevant for your study

Conflicts of interest are identified in the Conflicts of Interest section of the manuscript.

About the CONSORT EHEALTH checklist

As a result of using this checklist, did you make changes in your manuscript? *

- ○ yes, major changes
- ◉ yes, minor changes
- ○ no

What were the most important changes you made as a result of using this checklist?

Changes to the abstract and specific notes outlined in the checklist in order to conform to outlined recommendations/requirements.

How much time did you spend on going through the checklist INCLUDING making changes in your manuscript *

Approximately 2 hours in total

As a result of using this checklist, do you think your manuscript has improved? *

- ◉ yes
- ○ no
- ○ Other:

Would you like to become involved in the CONSORT EHEALTH group?

This would involve for example becoming involved in participating in a workshop and writing an "Explanation and Elaboration" document

- ○ yes
- ○ no
- ◉ Other: I would potentially be interesting depending on the time commitment

Clear selection

Any other comments or questions on CONSORT EHEALTH

Your answer

STOP - Save this form as PDF before you click submit

To generate a record that you filled in this form, we recommend to generate a PDF of this page (on a Mac, simply select "print" and then select "print as PDF") before you submit it.

When you submit your (revised) paper to JMIR, please upload the PDF as supplementary file.

Don't worry if some text in the textboxes is cut off, as we still have the complete information in our database. Thank you!

Final step: Click submit !

Click submit so we have your answers in our database!

Submit Clear form

Never submit passwords through Google Forms.

This form was created outside of your domain. Report Abuse - Terms of Service - Privacy Policy

Google Forms
